# Multidimensional Profiling of MRI‐Negative Temporal Lobe Epilepsy Uncovers Distinct Phenotypes

**DOI:** 10.1002/acn3.70349

**Published:** 2026-03-24

**Authors:** Alice Ballerini, Alessia Casarini, Niccolò Biagioli, Laura Mirandola, Daniela Ballotta, Paul Summers, Simona Scolastico, Laura Madrassi, Maurilio Genovese, Marcella Malagoli, Gaetano Cantalupo, Giada Giovannini, Matteo Pugnaghi, Niccolò Orlandi, Laura Tassi, Valeria Cuccarini, Domenico Aquino, Elena Tartara, Fulvia Palesi, Giuseppe Didato, Paolo Vitali, the 3TLE Study Group, Roberta Di Giacomo, Fabio Doniselli, Federica Mazzi, Carlo Andrea Galimberti, Claudia A. M. Gandini Wheeler‐Kingshott, Stefano Meletti, Anna Elisabetta Vaudano

**Affiliations:** ^1^ Department of Biomedical, Metabolic and Neural Sciences University of Modena and Reggio Emilia Modena Italy; ^2^ Neurology Unit San Giovanni Bosco Hospital Turin Italy; ^3^ ImageTech Labs Simon Fraser University Burnaby Canada; ^4^ Department of Radiology IEO European Institute of Oncology, IRCCS Milano Italy; ^5^ Diagnostic Imaging Villa Rosa Modena Italy; ^6^ Neuroradiology Unit AOU Modena Modena Italy; ^7^ Department of Engineering for Innovation Medicine University of Verona Verona Italy; ^8^ Center for Research on Epilepsy in Pediatric Age (CREP) and Child Neuropsychiatry Unit University Hospital of Verona Verona Italy; ^9^ Neuroscience Department Neurophysiology Unit and Epilepsy Centre, AOU Modena Italy; ^10^ “Claudio Munari” Epilepsy Surgery Center ASST Niguarda Hospital Milano Italy; ^11^ Neuroradiology Unit Fondazione IRCCS Istituto Neurologico Carlo Besta Milan Italy; ^12^ Epilepsy Center IRCCS Mondino Foundation Pavia Italy; ^13^ Department of Brain and Behavioral Sciences University of Pavia Pavia Italy; ^14^ Epilepsy Unit Fondazione IRCCS Istituto Neurologico Carlo Besta Milan Italy; ^15^ Department of Biomedical Science for Health State University of Milan Milan Italy

**Keywords:** amygdala enlargement, mri negative, temporal lobe epilepsy

## Abstract

**Objective:**

Although hippocampal sclerosis (TLE‐HS) represents the most frequent cause of temporal lobe epilepsy (TLE), up to 30% of patients show no lesion on visual MRI inspection (TLE‐MRIneg). These cases pose diagnostic and therapeutic challenges and are underrepresented in surgical series. We investigated whether TLE‐MRIneg constitutes a distinct clinical and neuroanatomical entity compared to TLE‐HS and aimed to identify subtypes within the TLE‐MRIneg group.

**Methods:**

We analyzed MRI and clinical data from 209 patients with TLE and 102 healthy controls from the multicenter “3TLE project”. Based on expert radiological review, 96 patients were classified as TLE‐MRIneg and 76 as TLE‐HS; the remaining 37 were excluded due to other focal lesions. We compared clinical characteristics and brain morphometry between TLE‐MRIneg and TLE‐HS and applied clustering techniques to detect TLE‐MRIneg subtypes.

**Results:**

Compared with TLE‐HS, TLE‐MRIneg was associated with later onset, shorter disease duration, and milder clinical presentation. TLE‐HS patients exhibited widespread cortical and subcortical atrophy, while TLE‐MRIneg showed only subtle cortical thinning. Cluster analysis revealed two subtypes of TLE‐MRIneg: one characterized by ipsilateral amygdala enlargement (AE) and the other by diffuse cortical atrophy.

**Interpretation:**

These findings demonstrate that TLE‐MRIneg represents a distinct clinical‐imaging entity from TLE‐HS. The identification of morphologically defined subtypes, particularly AE, highlights the heterogeneity of TLE‐MRIneg and its potential clinical relevance. This work supports the use of advanced imaging and data‐driven methods to improve diagnosis and guide individualized management in non‐lesional epilepsies.

**Abbreviations**
3D‐FLAIR: 3D fluid‐attenuated inversion recovery sequence
3D‐T1: 3D T1‐weighted sequence
AE: amygdala enlargement
ASMs: anti‐seizure medications
BLA: basolateral amygdala
DI: Dunn Index
FBTCS: focal to bilateral tonic–clonic seizure
FDR: false discovery rate
ICV: intracranial volume
LV: latent variable
MANCOVA: multivariate analysis of covariance
NNM: nearest neighbor matching
OCB: Baggiovara Academic Hospital
PCA: principal component analysis
PLS: partial least squares
ROI: region of interest
SD: standard deviation
SE: status epilepticus
TLE: temporal lobe epilepsy
TLE‐HS: temporal lobe epilepsy with hippocampal sclerosis
TLE‐MRIneg: temporal lobe epilepsy with negative MRI

## 1 Introduction

Temporal lobe epilepsy (TLE) is the most common form of focal epilepsy [1], representing a clinically and etiologically heterogeneous disorder that predominantly involves temporal lobe structures. While hippocampal sclerosis (TLE‐HS) is the most commonly identified cause, up to 30% of patients show no detectable lesions on MRI, even when dedicated epilepsy protocols are used [2, 3]. Non‐lesional TLE (TLE‐MRIneg) poses significant diagnostic and therapeutic challenges, since the lack of an identifiable epileptogenic lesion may preclude surgical consideration in some patients [4, 5, 6]. Recent studies have proposed radiological amygdala enlargement (AE) as a potential biomarker of TLE‐MRIneg patients [7, 8, 9, 10]. AE has been observed in 12%–63% of cases, but its epileptogenic and clinical significance remains controversial [11]. Advanced neuroimaging approaches, including artificial intelligence‐based analyses [12, 13], have made considerable efforts to detect cortical and subcortical biomarkers associated with TLE and to correlate these findings with clinical, demographic, and prognostic variables [14, 15, 16]. Among these, TLE‐HS has been thoroughly investigated and is well recognized as a syndrome with distinct clinical features and favorable surgical outcomes [17]. However, the characterization of TLE‐MRIneg remains particularly challenging and less well defined. Prior studies have combined TLE‐HS and TLE‐MRIneg, often based on the assumption of a hypothetical pathophysiological continuum [18, 19, 20, 21]. Drawing on evidence from previously published clinical investigations [22], we challenge this conceptual framework and propose instead that TLE‐MRIneg and TLE‐HS constitute distinct entities within TLE, each characterized by unique clinical and imaging profiles.

In this multicenter study, we seek to evaluate this hypothesis by employing a multimodal approach to identify clinical and neuroimaging phenotypes within TLE‐MRIneg patients. Acknowledging the heterogeneity among MRI‐negative cases may facilitate the development of targeted research strategies and foster advancements in personalized medicine for epilepsy.

## 2 Methods

### 2.1 Participant

A large multicenter cohort of patients with TLE was prospectively recruited between 2016 and 2020 from three Italian epilepsy centers: the Baggiovara Academic Hospital (OCB) of the University of Modena and Reggio Emilia, the IRCCS Mondino Neurological Institute (Pavia), and the IRCCS Carlo Besta Neurological Institute (Milan). Recruitment occurred within the framework of the national “3TLE Project” (NET‐2013‐02355313).

Clinical data and ictal semiology were collected for all patients through neurological examination, clinical history, routine EEG, and video‐EEG when available. TLE diagnoses were established according to the International League Against Epilepsy (ILAE) guidelines by board‐certified neurologists [23, 24]. Patients were classified as having left‐, right‐, or bilateral‐onset TLE based on consensus among clinicians (GD, LT, SM, AEV), integrating interictal and ictal EEG findings with seizure semiology. Based on the ictal semiology, neurophysiological information, and according to previously published evidence [25, 26, 27], TLE‐MRIneg patients were labeled as having a likely mesial or lateral neocortical temporal onset. All the collected clinical variables are summarized in Table 1.

**TABLE 1:** Clinical characteristics of TLE‐MRIneg and TLE‐HS patients.

	TLE	TLE‐MRIneg	TLE‐HS	Stat	Sign
*N*	172	96	76		
Age	42.16 (±14.872)	43.43 (±16.967)	40.55 (±11.615)	−1.261^t^	0.209
Sex (f/m)	98/73	55/41	43/33	0.009χ^2^	0.925
Age of onset	27.76 (±19.962)	34.19 (±20.708)	18.72 (±14.416)	−**5.535** ^t^	**< 0.001**
Duration	14.73 (±13.680)	9.45 (±10.922)	21.72 (±13.710)	**6.534** ^t^	**< 0.001**
Hemisphere					
Left temporal lobe	108	63	45	4.633χ^2^	0.099
Right temporal lobe	54	25	29		
Bitemporal	10	8	2		
Mesial (y/n)	155/17	79/17	76/0	**14.934**χ^2^	**< 0.001**
Frequency	3.35 (±1.371)	2.96 (±1.383)	3.85 (±1.186)	**4.494** ^t^	**< 0.001**
Number of ASMs at the MRI	1.87 (±0.924)	1.59 (±0.865)	2.23 (±0.879)	**4.713** ^t^	**< 0.001**
Number of ASMs in the past	1.23 (±1.664)	0.88 (±1.743)	1.69 (±1.442)	**3.282** ^t^	**0.001**
Drug‐resistance (y/n)	67/105	24/72	43/33	**17.789**χ^2^	**< 0.001**
Family history (y/n)^a^	31/136	18/75	13/61	0.087χ^2^	0.768
Febrile seizure (y/n)^b^	37/132	6/90	31/42	**31.805**χ^2^	**< 0.001**
Perinatal complications (y/n)^c^	15/156	7/89	8/67	0.599χ^2^	0.439
Head trauma (y/n)^c^	16/155	8/88	8/67	0.270χ^2^	0.603
Seizure‐free periods (y/n)^c^	57/114	31/65	26/49	0.107χ^2^	0.744
FBTCS (y/n)^c^	107/64	58/38	49/26	0.435χ^2^	0.510
Cluster and/or SE (y/n)^c^	48/123	19/77	30/46	**8.066**χ^2^	**0.005**
Falls related to seizure (y/n)^d^	40/130	12/83	28/47	**14.213**χ^2^	**< 0.001**
PNES (y/n)^c^	3/168	0/96	3/72	**3.909**χ^2^	**0.048**
Psychiatric disorders (y/n)^c^					
None	132	69	63	3.715χ^2^	0.156
Mixed anxiety‐depression	34	24	10		
Psychosis	5	3	2		
Neurosurgery (y/n)	43/129	4/92	39/37	**50.292**χ^2^	**< 0.001**
Slow‐wave activity (y/n)	128/44	71/25	57/19	0.024χ^2^	0.876
Epileptiform activity (y/n)	141/31	77/19	64/12	0.460χ^2^	0.498
Loss of awareness (y/n)^a^	63/104	44/50	19/54	**7.553**χ^2^	**0.006**
Seizure recall (y/n)^e^	53/107	33/56	20/51	1.415χ^2^	0.234
Aphasia (y/n)^f^	71/90	40/51	31/39	0.002χ^2^	0.967
Confusion (y/n)^e^	90/70	50/41	40/29	0.146χ^2^	0.702
Epigastric aura (y/n)^b^	69/100	31/64	38/36	**6.034**χ^2^	**0.014**
Auditory aura (y/n)^b^	11/158	8/87	3/71	1.304χ^2^	0.254
Visual aura (y/n)^b^	12/157	9/86	3/71	1.852χ^2^	0.174
Olfactory aura (y/n)^b^	6/163	5/90	1/73	1.859χ^2^	0.173
Gustatory aura (y/n)^b^	6/163	2/93	4/70	1.323χ^2^	0.250
Autonomic aura (y/n)^b^	64/105	42/53	22/52	3.707χ^2^	0.054
Psychic aura (y/n)^b^	72/97	38/57	34/40	0.601χ^2^	0.438
Unilateral sensory‐motor aura (y/n)^b^	8/161	4/91	4/70	0.132χ^2^	0.717
Bilateral sensory‐motor aura (y/n)^b^	5/164	1/94	4/70	2.745χ^2^	0.098
Oral automatism (y/n)^g^	64/100	23/68	41/32	**16.243**χ^2^	**< 0.001**
Unilateral manual automatism (y/n)^h^	27/138	12/80	15/58	1.675χ^2^	0.196
Bilateral manual automatism (y/n)^g^	23/141	10/81	13/60	1.562χ^2^	0.211
Hyperkinetic automatism (y/n)^g^	6/158	5/86	1/72	1.955χ^2^	0.162

*Note:* Age, age at epilepsy onset, duration of epilepsy, seizure frequency, and number of antiseizure medications (ASMs) are presented as mean (± standard deviation). Age‐related variables are reported in years (y). Seizure frequency is categorized as follows: (1) < 1 seizure/year, (2) 1–3/year, (3) 4–11/year, (4) 1–3/month, (5) 1–6/week, (6) 1–3/day, and (7) > 4/day. Categorical variables are coded as “yes” (y) or “no” (n), while sex is reported as “female” (f) or “male” (m). Statistical tests include independent samples *t*‐tests (*t*) and chi‐square tests (χ^2^). Level of consciousness was defined by the presence of impaired awareness and post‐ictal recall. Missing data are as follows: ^a^5, ^b^3, ^c^1, ^d^2, ^e^12, ^f^11, ^g^8, and ^h^7. The first column summarizes data for the full TLE cohort; subsequent columns present group comparisons between TLE‐MRIneg and TLE‐HS patients. Significance levels are reported as *p*‐values; *p* < 0.05 are highlighted in bold.

Abbreviations: Stat, statistics; Sign, significance; FBTCS, focal to bilateral tonic–clonic seizure; PNES, psychogenic non‐epileptic seizure; SE, status epilepticus.

**FIGURE 1:**
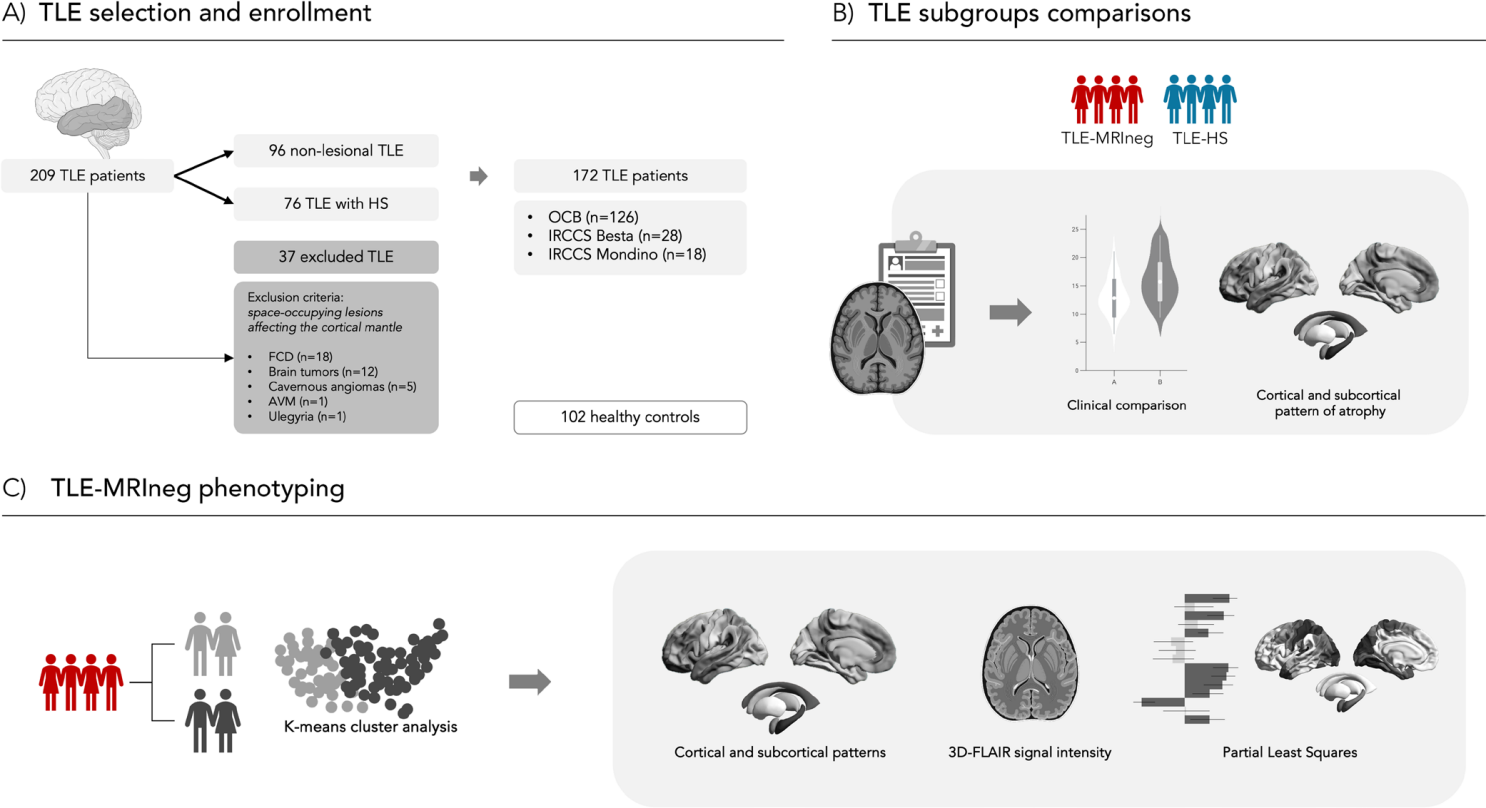
Study flow diagram and patient selection. (A) Overview of the selection process for patients with temporal lobe epilepsy (TLE) across centers, including exclusion criteria and the final cohort. (B) Illustration of the clinical and neuroanatomical analyses conducted between TLE patients with MRI‐negative (TLE‐MRIneg) and with hippocampal sclerosis (TLE‐HS). (C) Phenotyping of the TLE‐MRIneg group through *k*‐means clustering, analysis of cortico‐subcortical structural patterns, 3D‐FLAIR signal intensity, and partial least squares analysis. AVM, arteriovenous malformation; FCD, focal cortical dysplasia; OCB, Baggiovara Academic Hospital.

All patients underwent a high‐resolution 3T structural MRI according to the HARNESS protocol [28], including an isotropic 3D T1‐weighted sequence (3D‐T1) and a 3D fluid‐attenuated inversion recovery (3D‐FLAIR) sequence. In the present study, patients with HS [29] and patients with negative MRI findings were included, while individuals with other focal lesions were excluded (see the study flowchart, Figure 1). Patient classification as TLE‐HS or TLE‐MRIneg was based on expert visual MRI assessment (MG, MM), consistent with real‐world clinical practice.

Finally, a large age‐ and sex‐matched healthy volunteer cohort with no history of neurological or psychiatric illness, who underwent the same MRI protocol, was recruited from OCB Academic Hospital.

The study was approved by the Ethical Committee of Area Vasta Emilia Nord (N. 312/2015 and N. 679/2022) and the Ethical Committee of Regione Lombardia (N. 24/2015). Patients gave written informed consent for the use of their clinical records for this study. The study was conducted in accordance with the World Medical Association Declaration of Helsinki.

### 2.2 Image Processing

The *3D‐T1 sequences* were processed using FreeSurfer [30] (v7.3.2) to compute cortical thickness and subcortical volumetric measures. Subject‐specific cortical surface maps were reconstructed and registered to the Conte69 template [31, 32], with cortical thickness estimated at 32,000 vertices per hemisphere. Subcortical segmentation yielded volumetric estimates for 16 structures. Dedicated FreeSurfer pipelines were employed to subsegment the hippocampal subfields [33], amygdala nuclei [34], and thalamic nuclei [35]. All segmentation and cortical reconstructions were subjected to a quality control procedure following standardized ENIGMA protocols. Since the neuroimaging evaluation was conducted on different 3T scanners (Table S1), all the brain features were harmonized using neuroCombat [36].

The *3D‐FLAIR sequences* were inspected quantitatively to detect subtle mesial temporal signal alterations overlooked on visual inspection in the TLE‐MRIneg group only. FLAIR images were preprocessed with skull stripping, bias‐field correction using the N4 algorithm [37], and registration to the individual 3D‐T1. Mean signal intensity was extracted from subject‐specific hippocampal and amygdalae masks derived from FreeSurfer segmentation. To account for inter‐scanner and inter‐protocol variability in this multicenter cohort, FLAIR signal intensities were standardized using a basal ganglion–based normalization approach [38].

Hippocampal volume and quantitative FLAIR signal intensity were inspected at the individual level to complement expert neuroradiological evaluation and to identify hippocampal abnormalities not detected by visual inspection alone. Hippocampal atrophy was defined as a volumetric value ≤ −2 standard deviations (SDs) relative to the healthy control distribution in at least one hippocampal subregion (head, body, or tail), whereas 3D‐FLAIR hyperintensity was defined as a signal intensity ≥ +1.5 SDs relative to controls.

More details on image pre‐processing are fully described in the Supporting Informations.

### 2.3 Brain Morphometry Analysis and Nearest Neighbor Matching

Harmonized brain features were converted into z‐scores based on the healthy controls' mean and SD. Cortical and subcortical values of right‐lateralized patients were flipped to ensure that all morphometric data were ipsilateral to the epileptic focus in the left hemisphere [7, 19, 39]. Since z‐scoring was performed relative to the control group's distribution before side alignment, potential physiological left and right asymmetries were inherently taken into account. However, we performed an additional paired‐sample *t*‐test on controls' z‐scores to assess potential asymmetries in the hippocampal and amygdalae formations.

Cortical thickness was analyzed using a vertex‐wise approach to surface maps via BrainStat [40]. First, we examined the morphometric cortico‐subcortical differences between the entire TLE population and the controls, followed by comparisons between TLE‐MRIneg, TLE‐HS, and the controls. In all comparisons, age and sex were included as covariates. Similarly, multivariate analyses of covariance (MANCOVAs) were conducted to assess volumetric differences in subcortical structures and sub‐segmentations across groups, including age, sex, and intracranial volume (ICV) as covariates. Statistical significance was set at *p* < 0.05, and *p*‐values were adjusted using false discovery rate (FDR) [41].

Secondly, to minimize the potential confounding effects of age at seizure onset and disease duration on morphometric results, we performed a post hoc 1:1 nearest neighbor matching (NNM) [42, 43]. Each TLE‐MRIneg patient was individually matched to the TLE‐HS patient with the most similar age of onset and epilepsy duration. After matching, the morphometric and clinical analyses were repeated as before.

### 2.4 Cluster Analysis

We applied *k*‐means cluster analysis [44] to the TLE‐MRIneg cohort (Figure 1C) to improve their classification and characterization. To mitigate overfitting due to the high number of vertices, cortical surfaces were down‐sampled into 360 cortical regions of interest (ROIs) according to the multimodal Glasser atlas [45] using the “surface to parcel” function from the ENIGMA‐Toolbox [46]. The optimal number of clusters was determined using Thorndike's “elbow method” [47]. Principal component analysis (PCA) was applied to the z‐scored cortico‐subcortical data, then *k*‐means clustering was performed on the selected PCA components using the MATLAB “kmeans” function. To assess clustering quality, the Dunn Index (DI) was computed [48]. Potential outliers were identified and controlled before finalizing the clustering. Once all patients were assigned to clusters, clinical differences between TLE‐MRIneg clusters and TLE‐HS were assessed. We ensured no significant differences in age and sex distribution between clusters and controls by 1:1 NNM, then morphometric comparisons were performed as previously described.

Finally, signal intensity analyses of the hippocampi and amygdalae from the 3D‐FLAIR were performed between the identified TLE‐MRIneg clusters and healthy controls using analysis of covariance (ANCOVA), with age and sex included as covariates.

### 2.5 Clinical Phenotypes Associated With Brain Morphometric Features

Within each cluster, we performed Pearson correlations between epilepsy clinical variables and brain ROIs that significantly differed from controls (see Supporting Informations).

Subsequently, partial least squares (PLS) analysis was performed within each cluster to identify clinical variable combinations that best explained the observed morphometric patterns. To reduce dimensionality and avoid overfitting, clinical variables were aggregated into composite scores, and cortical data were summarized using Glasser parcellation. Brain and clinical data were correlated using the “myPLS” MATLAB toolbox [49, 50]. Model significance was assessed via permutation testing (*N* = 10,000) [51], while bootstrap resampling (*N* = 10,000) estimated confidence intervals [52]. Bootstrap ratio values ±1.96 identified the most stable contributing variables. Cross‐validation was conducted using a 4‐fold approach with 100 randomized train‐test splits (75/25%) and a leave‐one‐out method to assess out‐of‐sample performance. The reported permutation *p*‐value (*P*_perm_) reflects the average *p*‐value derived from the permutation distributions across the 100 cross‐validation iterations. Further methodological details are reported elsewhere [14, 53], and additional information about variable selection and model optimization is provided in the Supporting Informations and Figure S1.

## 3 Results

### 3.1 Patients and Clinical Profiles

Structural MRI scans were collected from 209 patients and 102 controls. The visual radiological assessment classified 96 patients as TLE‐MRIneg, 76 as TLE‐HS, and 37 patients were excluded due to the presence of other focal lesions (Figure 1A).

TLE‐HS and TLE‐MRIneg patients did not differ from controls in terms of age or sex distribution (Table S2). A detailed comparison of clinical and electro‐clinical features of each cohort is presented in Table 1. TLE‐MRIneg patients had an older age at epilepsy onset and a shorter disease duration, along with a milder clinical profile characterized by lower seizure frequency, fewer anti‐seizure medications (ASMs), and reduced drug refractoriness compared with TLE‐HS. They also exhibited a lower prevalence of febrile seizures in childhood and were less likely to have a history of seizure clusters or status epilepticus (SE). Moreover, TLE‐MRIneg patients reported fewer epigastric auras, oral automatisms, and episodes of impaired awareness during seizures. Within the TLE‐MRIneg group, 79 of 96 patients were identified as having a likely mesial onset, whereas 17 were classified as lateral or of uncertain lateralization. As expected, a smaller proportion of TLE‐MRIneg patients underwent pre‐surgical evaluation and epilepsy surgery compared with TLE‐HS. Indeed, only four TLE‐MRIneg patients underwent anterior temporal lobectomy; histopathological examination revealed gliosis in three cases, whereas one patient showed no abnormalities. The individual volumetric and signal intensities assessment revealed that 16 TLE‐MRIneg patients (17%) showed evidence of hippocampal abnormalities. Specifically, six patients exhibited atrophy in at least one main hippocampal subfield (i.e., head, body, or tail), and six patients showed 3D‐FLAIR hyperintensity in at least one hippocampus. Only four patients (4%) fulfilled both criteria, with hippocampal volume reduction and signal increase (Figure S2).

### 3.2 Cortico‐Subcortical Patterns of Atrophy

**FIGURE 2:**
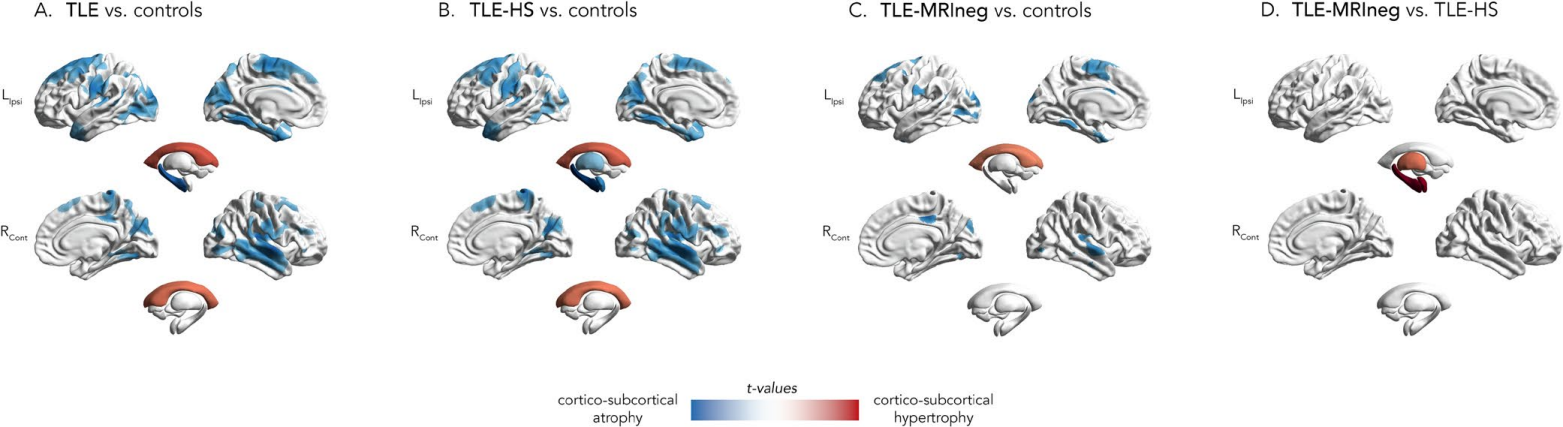
Whole‐brain comparisons between TLE patients and healthy controls. The figure illustrates group differences in cortical thickness and subcortical volumes between healthy controls and patients with temporal lobe epilepsy (TLE). Panel A depicts the comparison with the entire TLE cohort, panel B shows patients with hippocampal sclerosis (TLE‐HS), and panel C shows patients with MRI‐negative TLE (TLE‐MRIneg). Finally, panel D presents the direct comparison between the two TLE subgroups (TLE‐MRIneg vs. TLE‐HS). Only regions showing statistically significant effects after false discovery rate (FDR) correction (*P*_FDR_ < 0.05) are shown. All comparisons were adjusted for age and sex, and subcortical comparisons were additionally adjusted for intracranial volume (ICV). Brain maps represent t‐values and were generated using the ENIGMA Toolbox [46].

Although minor physiological asymmetries were observed in healthy controls, with slightly larger right‐sided hippocampal and amygdalae volumes, these differences were not statistically significant (Table S3). TLE‐MRIneg (*P*_FDR_ = 0.004) and TLE‐HS (*P*_FDR_ = 0.006) showed lower ICV compared to controls (Table S2). When examining cortico‐subcortical patterns of atrophy, TLE‐HS exhibited the same widespread bilateral cortical thinning and reduced ipsilateral hippocampal volume observed in the overall TLE cohort (Figure 2A). In contrast, TLE‐MRIneg showed more limited cortical thinning (Figure 2A), primarily affecting the ipsilateral precentral gyrus and the basal portions of the mesial temporal lobe, regions also affected in TLE‐HS. No significant subcortical volume differences were found between TLE‐MRIneg patients and controls (Table S4), except for an enlargement of the ipsilateral lateral ventricle (*P*_FDR_ = 0.037). These findings suggested that the pathological profile of the TLE‐HS subgroup primarily drives the atrophy pattern observed in the entire TLE population. Although no differences in cortical thickness were detected when directly comparing TLE‐HS and TLE‐MRIneg, significant subcortical atrophy of the ipsilateral hippocampus, amygdala, and thalamus confirmed the distinct pathology likely underlying the two TLE subgroups (Figure 2). Details on hippocampal subfields analyses are provided in Table S4, together with further evidence of additional atrophy in the ipsilateral amygdala, thalamus, and their substructures in TLE‐HS when compared to the TLE‐MRIneg cohort. Unthreshold *t*‐value brain maps are shown in Figure S3.

**FIGURE 3:**
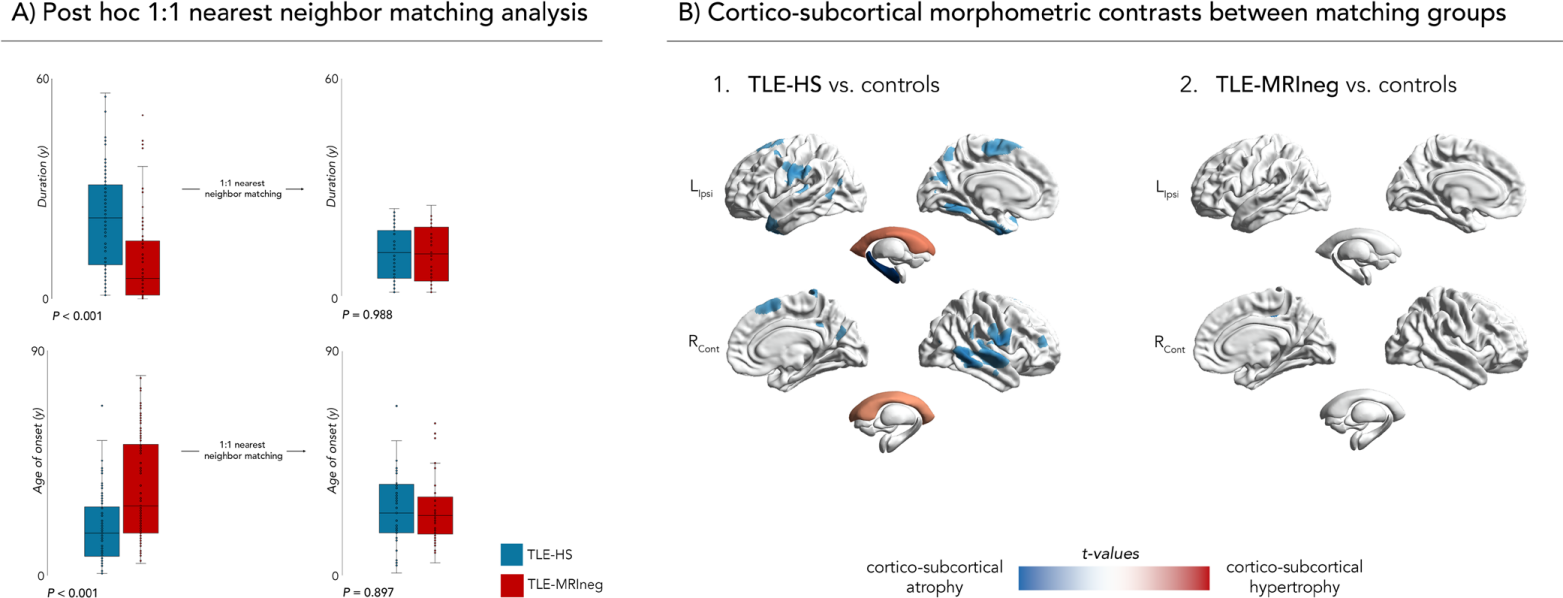
Whole‐brain comparisons after matching TLE‐HS and TLE‐MRIneg for age at onset and epilepsy duration. The figure shows the post hoc analyses comparing brain morphometry between patients with temporal lobe epilepsy (TLE) with hippocampal sclerosis (TLE‐HS) and MRI‐negative findings (TLE‐MRIneg) and healthy controls after 1:1 nearest‐neighbor matching (NNM) for age at epilepsy onset and disease duration. Box‐and‐whisker plots in panel A illustrate the distributions before (left) and after (right) matching. Central lines indicate the median, boxes represent the interquartile range, and dots correspond to individual patients. Panel B depicts whole‐brain comparisons after 1:1 NNM between controls and TLE‐HS (B1) and controls and TLE‐MRIneg (B2). Only regions showing statistically significant effects after false discovery rate (FDR) correction (*P*_FDR_ < 0.05) are shown. All comparisons were adjusted for age and sex, and subcortical comparisons were additionally adjusted for intracranial volume (ICV). Brain maps represent t‐values and were generated using the ENIGMA Toolbox [46].

To ensure that the observed MRI patterns were not merely driven by differences in age of onset or epilepsy duration, we employed a 1:1 NNM approach to control for the marked differences in age at epilepsy onset and disease duration between TLE‐MRIneg and TLE‐HS (Figure 3A). With this procedure, we confirmed observed patterns of cortico‐subcortical atrophy in TLE‐HS relative to controls, while TLE‐MRIneg did not differ in cortical–subcortical patterns compared to controls (Figure 3B). Clinical comparisons within the matched subpopulation confirmed the group differences between TLE‐MRIneg and TLE‐HS previously reported in the full cohort analysis (Table S5).

Finally, we performed morphometric analyses restricted to the suspected mesial TLE‐MRIneg cohort, and the results confirmed the absence of cortical and subcortical mesial temporal abnormalities. Specifically, hippocampal and amygdalae volumes in the mesial subgroup were comparable to those of healthy controls and larger than TLE‐HS patients, both at the whole‐structure and subfield levels. Similar findings were observed for the thalamus and its subnuclei. All results are provided in Figure S4 and Table S6.

### 3.3 MRI‐Negative TLE: Clinical and Morphometric Phenotyping

Based on cortical and subcortical morphometric features, *k*‐means clustering identified two clusters within the TLE‐MRIneg population (Figure S5). After confirming good clustering quality (DI = 0.361) and the absence of potential outliers, the TLE‐MRIneg group was subdivided into Cluster 1 (*N* = 53) and Cluster 2 (*N* = 43).

Clinical comparisons between the two Clusters and between the Clusters and TLE‐HS are presented in Table S7. Notably, patients in Cluster 2 were significantly older with later epilepsy onset compared with Cluster 1. Differing from Cluster 1, Cluster 2 showed similar lifetime ASMs use, seizure frequency, and SE history to TLE‐HS, suggesting suboptimal seizure control and greater therapeutic complexity.

**FIGURE 4:**
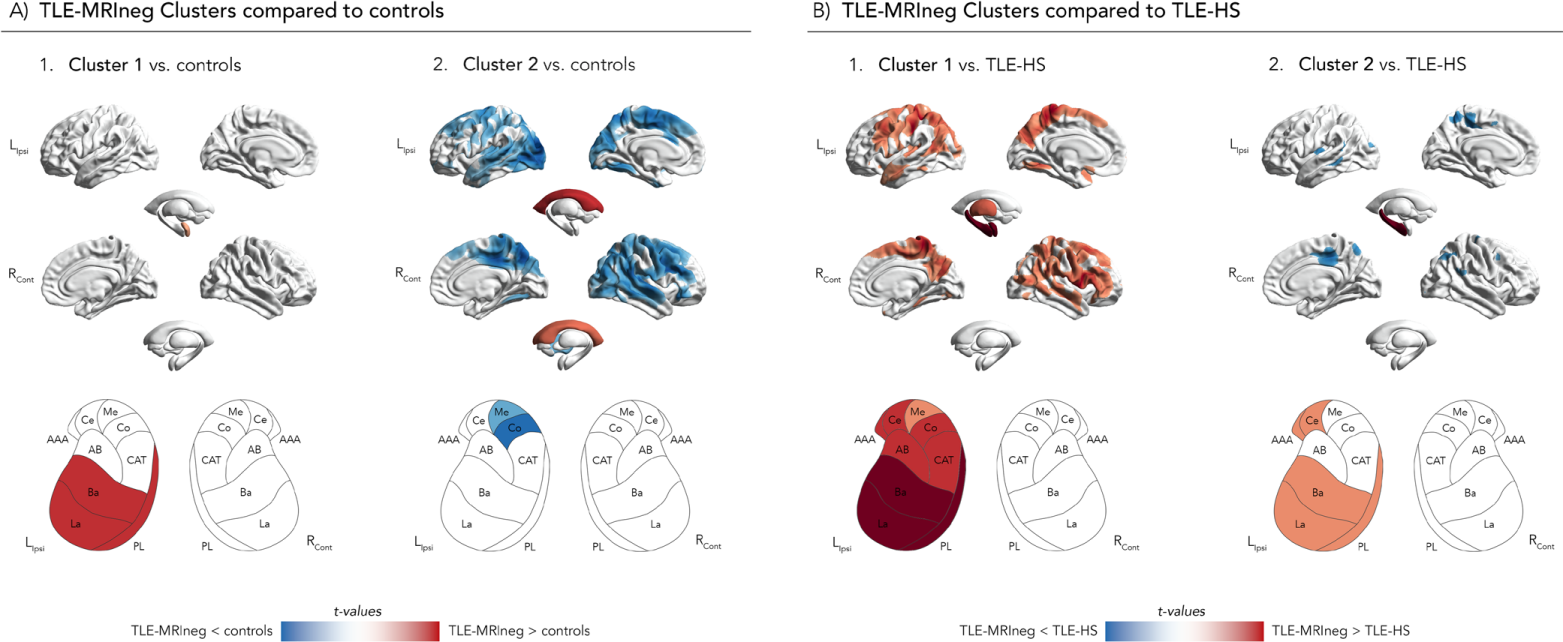
Cortico‐subcortical and amygdala subnuclei patterns across TLE‐MRIneg clusters. The figure illustrates whole‐brain comparisons between MRI‐negative temporal lobe epilepsy (TLE‐MRIneg) Cluster 1 and Cluster 2, and both healthy controls and patients with hippocampal sclerosis (TLE‐HS). Panel A shows differences between healthy controls and TLE‐MRIneg Cluster 1 (A1) and Cluster 2 (A2). Panel B depicts differences between TLE‐HS and TLE‐MRIneg Cluster 1 (B1) and Cluster 2 (B2). Only regions with statistically significant differences after false discovery rate (FDR) correction (*P*_FDR_ < 0.05) are shown. All comparisons were adjusted for age and sex, and subcortical comparisons were additionally adjusted for intracranial volume (ICV). Brain maps represent t‐values and were generated using the ENIGMA Toolbox [46].

From a morphometric perspective, Cluster 1 showed increased ipsilateral amygdala volume, mainly involving the basolateral amygdala (BLA; Figure 4A and Table 2), compared with healthy controls. In contrast, Cluster 2 showed global cortical atrophy, reduced ICV (t = 2.517, *p* = 0.014), and ipsilateral atrophy in the medial and cortical amygdala nuclei (Figure 4A and Table 3). Unthreshold t‐value brain maps are shown in Figure S6.

**TABLE 2:** Subcortical volume comparisons between TLE‐MRIneg Cluster 1, TLE‐HS, and healthy controls.

	MANCOVA results	TLE‐MRIneg Cluster 1 vs. controls	TLE‐MRIneg Cluster 1 vs. TLE‐HS
Ipsilateral hippocampus			
Subiculum	**F**_**(1,153)**_ **= 33.747, *p* < 0.001**	*t* = 0.931, *P*_FDR_ = 0.977	***t* = 7.557, *P***_**FDR**_ < **0.001**
Presubiculum	**F**_**(1,153)**_ **= 34.789, *p* < 0.001**	*t* = 2.242, *P*_FDR_ = 0.195	***t* = 8.093, *P***_**FDR**_ < **0.001**
Parasubiculum	**F**_**(1,153)**_ **= 5.901, *p* = 0.003**	*t* = 2.254, *P*_FDR_ = 0.195	***t* = 3.369, *P***_**FDR**_ **= 0.001**
CA1	**F**_**(1,153)**_ **= 37.157, *p* < 0.001**	*t* = −0.466, *P*_FDR_ = 0.977	***t* = 7.250, *P***_**FDR**_ < **0.001**
CA3	**F**_**(1,153)**_ **= 24.357, *p* < 0.001**	*t* = −0.241, *P*_FDR_ = 0.977	***t* = 5.949, *P***_**FDR**_ < **0.001**
CA4	**F**_**(1,153)**_ **= 44.385, *p* < 0.001**	*t* = −0.086, *P*_FDR_ = 0.983	***t* = 8.146, *P***_**FDR**_ < **0.001**
Dental gyrus	**F**_**(1,153)**_ **= 43.252, *p* < 0.001**	*t* = −0.021, *P*_FDR_ = 0.983	***t* = 8.075, *P***_**FDR**_ < **0.001**
Molecular layer	**F**_**(1,153)**_ **= 47.184, *p* < 0.001**	*t* = 0.308, *P*_FDR_ = 0.977	***t* = 8.593, *P***_**FDR**_ < **0.001**
Hippocampal fissure	F_(1,153)_ = 1.506, *p* = 0.225	*t* = −1.648, *P*_FDR_ = 0.505	*t* = −0.343, *P*_FDR_ = 0.732
Fimbria	**F**_**(1,153)**_ **= 9.253, *p* < 0.001**	*t* = 1.215, *P*_FDR_ = 0.848	***t* = 4.187, *P***_**FDR**_ < **0.001**
HATA	**F**_**(1,153)**_ **= 4.747, *p* = 0.010**	*t* = 0.203, *P*_FDR_ = 0.977	***t* = 2.775, *P***_**FDR**_ **= 0.006**
Body	**F**_**(1,153)**_ **= 48.198, *p* < 0.001**	*t* = 0.816, *P*_FDR_ = 0.977	***t* = 8.907, *P***_**FDR**_ **< 0.001**
Head	**F**_**(1,153)**_ **= 37.183, *p* < 0.001**	*t* = 0.194, *P*_FDR_ = 0.977	***t* = 7.590, *P***_**FDR**_ **< 0.001**
Tail	**F**_**(1,153)**_ **= 29.463, *p* < 0.001**	*t* = 0.641, *P*_FDR_ = 0.977	***t* = 6.966, *P***_**FDR**_ **< 0.001**
Whole hippocampus	**F**_**(1,153)**_ **= 47.092, *p* < 0.001**	*t* = 0.482, *P*_FDR_ = 0.977	***t* = 8.665, *P***_**FDR**_ **< 0.001**
Ipsilateral amygdala			
Lateral nucleus	**F**_**(1,153)**_ **= 20.180, *p* < 0.001**	***t* = 2.905, *P***_**FDR**_ **= 0.042**	***t* = 6.348, *P***_**FDR**_ **< 0.001**
Basal nucleus	**F**_**(1,153)**_ **= 18.629, *p* < 0.001**	***t* = 2.621, *P***_**FDR**_ **= 0.042**	***t* = 6.089, *P***_**FDR**_ **< 0.001**
AB nucleus	**F**_**(1,153)**_ **= 11.668, *p* < 0.001**	*t* = 1.258, *P*_FDR_ = 0.315	***t* = 4.676, *P***_**FDR**_ **< 0.001**
AAA	**F**_**(1,153)**_ **= 17.676, *p* < 0.001**	*t* = 1.977, *P*_FDR_ = 0.100	***t* = 5.850, *P***_**FDR**_ **< 0.001**
Central nucleus	**F**_**(1,153)**_ **= 8.644, *p* < 0.001**	*t* = 0.787, *P*_FDR_ = 0.576	***t* = 3.940, *P***_**FDR**_ **< 0.001**
Medial nucleus	**F**_**(1,153)**_ **= 4.575, *p* = 0.012**	*t* = −0.563, *P*_FDR_ = 0.689	***t* = 2.307, *P***_**FDR**_ **= 0.022**
Cortical nucleus	**F**_**(1,153)**_ **= 9.429, *p* < 0.001**	*t* = −0.207, *P*_FDR_ = 0.893	***t* = 3.669, *P***_**FDR**_ **< 0.001**
CAT	**F**_**(1,153)**_ **= 9.963, *p* < 0.001**	*t* = 1.807, *P*_FDR_ = 0.125	***t* = 4.441, *P***_**FDR**_ **< 0.001**
Paralaminar nucleus	**F**_**(1,153)**_ **= 18.712, *p* < 0.001**	***t* = 2.548, *P***_**FDR**_ **= 0.042**	***t* = 6.094, *P***_**FDR**_ **< 0.001**
BLA	**F**_**(1,153)**_ **= 19.665, *p* < 0.001**	***t* = 2.489, *P***_**FDR**_ **= 0.042**	***t* = 6.235, *P***_**FDR**_ **< 0.001**
CMA	**F**_**(1,153)**_ **= 7.624, *p* = 0.001**	*t* = 0.135, *P*_FDR_ = 0.893	***t* = 3.461, *P***_**FDR**_ **= 0.001**
Whole amygdala	**F**_**(1,153)**_ **= 19.185, *p* < 0.001**	***t* = 2.383, *P***_**FDR**_ **= 0.043**	***t* = 6.150, *P***_**FDR**_ **< 0.001**
Ipsilateral thalamus			
Anterior	**F**_**(1,153)**_ **= 5.882, *p* = 0.004**	*t* = 0.795, *P*_FDR_ = 0.428	***t* = 3.275, *P***_**FDR**_ **= 0.002**
Lateral	**F**_**(1,153)**_ **= 6.791, *p* = 0.001**	*t* = 1.339, *P*_FDR_ = 0.255	***t* = 3.650, *P***_**FDR**_ **< 0.001**
Ventral	**F**_**(1,153)**_ **= 4.337, *p* = 0.015**	*t* = 2.103, *P*_FDR_ = 0.179	***t* = 2.828, *P***_**FDR**_ **= 0.007**
Intralaminar	**F**_**(1,153)**_ **= 3.620, *p* = 0.029**	*t* = 1.823, *P*_FDR_ = 0.179	***t* = 2.622, *P***_**FDR**_ **= 0.010**
Medial	**F**_**(1,153)**_ **= 7.322, *p* = 0.001**	*t* = 1.643, *P*_FDR_ = 0.179	***t* = 3.819, *P***_**FDR**_ **< 0.001**
Posterior	**F**_**(1,153)**_ **= 3.878, *p* = 0.023**	*t* = 0.872, *P*_FDR_ = 0.428	***t* = 2.730, *P***_**FDR**_ **= 0.008**
Whole thalamus	**F**_**(1,153)**_ **= 6.340, *p* = 0.002**	*t* = 1.664, *P*_FDR_ = 0.179	***t* = 3.559, *P***_**FDR**_ **< 0.001**

*Note:* The table reports results from MANCOVAs examining the ipsilateral subcortical structures (i.e., hippocampus, amygdala, and thalamus) and their sub‐segmentations. Significance levels are reported as *p*‐values adjusted for multiple comparisons using the false discovery rate (*P*_FDR_); *P*_FDR_ < 0.05 are highlighted in bold.

Abbreviations: AAA, anterior amygdaloid area; AB, accessory‐basal nuclei; BLA, basolateral amygdala (including lateral, basal, AB, and paralaminar nuclei); CAT, cortico‐amygdaloid transition area; CMA, central‐medial amygdala (including central and medial nuclei); HATA, hippocampal‐amygdaloid transition area.

**TABLE 3:** Subcortical volume comparisons between TLE‐MRIneg Cluster 2, TLE‐HS, and healthy controls.

	MANCOVA results	TLE‐MRIneg Cluster 2 vs. controls	TLE‐MRIneg Cluster 2 vs. TLE‐HS
Ipsilateral hippocampus			
Subiculum	**F**_**(1,123)**_ **= 22.946, *p* < 0.001**	*t* = −0.354, *P*_FDR_ = 0.724	***t* = 5.686, *P***_**FDR**_ **< 0.001**
Presubiculum	**F**_**(1,123)**_ **= 22.685, *p* < 0.001**	*t* = −0.384, *P*_FDR_ = 0.702	***t* = 5.638, *P***_**FDR**_ **< 0.001**
Parasubiculum	**F**_**(1,123)**_ **= 7.092, *p* = 0.001**	*t* = 0.776, *P*_FDR_ = 0.439	***t* = 3.582, *P***_**FDR**_ **< 0.001**
CA1	**F**_**(1,123)**_ **= 32.606, *p* < 0.001**	*t* = −1.397, *P*_FDR_ = 0.165	***t* = 6.198, *P***_**FDR**_ **< 0.001**
CA3	**F**_**(1,123)**_ **= 19.540, *p* < 0.001**	*t* = −0.184, *P*_FDR_ = 0.854	***t* = 5.325, *P***_**FDR**_ **< 0.001**
CA4	**F**_**(1,123)**_ **= 33.374, *p* < 0.001**	*t* = −0.454, *P*_FDR_ = 0.651	***t* = 6.842, *P***_**FDR**_ **< 0.001**
Dental gyrus	**F**_**(1,123)**_ **= 33.015, *p* < 0.001**	*t* = −0.556, *P*_FDR_ = 0.579	***t* = 6.749, *P***_**FDR**_ **< 0.001**
Molecular layer	**F**_**(1,123)**_ **= 34.972, *p* < 0.001**	*t* = −1.041, *P*_FDR_ = 0.300	***t* = 6.672, *P***_**FDR**_ **< 0.001**
Hippocampal fissure	F_(1,123)_ = 0.629, *p* = 0.535	*t* = −1.062, *P*_FDR_ = 0.290	*t* = −0.217, *P*_FDR_ = 0.829
Fimbria	F_(1,123)_ = 0.937, *p* = 0.395	*t* = −0.805, *P*_FDR_ = 0.422	*t* = 0.558, *P*_FDR_ = 0.619
HATA	F_(1,123)_ = 1.048, *p* = 0.354	*t* = 0.283, *P*_FDR_ = 0.778	*t* = 1.372, *P*_FDR_ = 0.200
Body	**F**_**(1,123)**_ **= 33.653, *p* < 0.001**	*t* = −0.352, *P*_FDR_ = 0.726	***t* = 6.930, *P***_**FDR**_ **< 0.001**
Head	**F**_**(1,123)**_ **= 25.910, *p* < 0.001**	*t* = −1.107, *P*_FDR_ = 0.271	***t* = 5.612, *P***_**FDR**_ **< 0.001**
Tail	**F**_**(1,123)**_ **= 24.480, *p* < 0.001**	*t* = −1.503, *P*_FDR_ = 0.135	***t* = 5.172, *P***_**FDR**_ **< 0.001**
Whole hippocampus	**F**_**(1,123)**_ **= 33.123, *p* < 0.001**	*t* = −1.092, *P*_FDR_ = 0.277	***t* = 6.445, *P***_**FDR**_ **< 0.001**
Ipsilateral amygdala			
Lateral nucleus	**F**_**(1,123)**_ **= 5.888, *p* = 0.004**	*t* = −0.750, *P*_FDR_ = 0.652	***t* = 2.527, *P***_**FDR**_ **= 0.046**
Basal nucleus	**F**_**(1,123)**_ **= 4.295, *p* = 0.016**	*t* = −0.284, *P*_FDR_ = 0.777	***t* = 2.377, *P***_**FDR**_ **= 0.046**
AB nucleus	F_(1,123)_ = 2.800, *p* = 0.065	*t* = −1.327, *P*_FDR_ = 0.521	*t* = 1.035, *P*_FDR_ = 0.403
AAA	**F**_**(1,123)**_ **= 6.887, *p* = 0.001**	*t* = −1.242, *P*_FDR_ = 0.521	***t* = 2.412, *P***_**FDR**_ **= 0.046**
Central nucleus	**F**_**(1,123)**_ **= 5.867, *p* = 0.004**	*t* = −0.655, *P*_FDR_ = 0.652	***t* = 2.588, *P***_**FDR**_ **= 0.046**
Medial nucleus	**F**_**(1,123)**_ **= 5.351, *p* = 0.006**	***t* = −2.778, *P***_**FDR**_ **= 0.036**	*t* = 0.111, *P*_FDR_ = 0.912
Cortical nucleus	**F**_**(1,123)**_ **= 6.455, *p* = 0.002**	***t* = −3.001, *P***_**FDR**_ **= 0.036**	*t* = 0.214, *P*_FDR_ = 0.907
CAT	F_(1,123)_ = 1.024, *p* = 0.362	*t* = −0.611, *P*_FDR_ = 0.652	*t* = 0.817, *P*_FDR_ = 0.498
Paralaminar nucleus	**F**_**(1,123)**_ **= 5.320, *p* = 0.006**	*t* = −0.338, *P*_FDR_ = 0.777	***t* = 2.642, *P***_**FDR**_ **= 0.046**
BLA	**F**_**(1,123)**_ **= 4.772, *p* = 0.010**	*t* = −0.747, *P*_FDR_ = 0.652	*t* = 2.225, *P*_FDR_ = 0.056
CMA	**F**_**(1,123)**_ **= 5.783, *p* = 0.004**	*t* = −1.904, *P*_FDR_ = 0.236	*t* = 1.492, *P*_FDR_ = 0.207
Whole amygdala	**F**_**(1,123)**_ **= 4.783, *p* = 0.010**	*t* = −0.935, *P*_FDR_ = 0.652	*t* = 2.090, *P*_FDR_ = 0.067
Ipsilateral thalamus			
Anterior	**F**_**(1,123)**_ **= 4.446, *p* = 0.014**	*t* = −1.758, *P*_FDR_ = 0.151	*t* = 1.210, *P*_FDR_ = 0.228
Lateral	**F**_**(1,123)**_ **= 6.018, *p* = 0.003**	*t* = −2.617, *P*_FDR_ = 0.056	*t* = 0.672, *P*_FDR_ = 0.503
Ventral	F_(1,123)_ = 1.727, *p* = 0.182	*t* = −0.770, *P*_FDR_ = 0.443	*t* = 1.082, *P*_FDR_ = 0.281
Intralaminar	**F**_**(1,123)**_ **= 3.141, *p* = 0.047**	*t* = −1.530, *P*_FDR_ = 0.151	*t* = 0.957, *P*_FDR_ = 0.340
Medial	**F**_**(1,123)**_ **= 6.775, *p* = 0.002**	*t* = −2.446, *P*_FDR_ = 0.056	*t* = 1.162, *P*_FDR_ = 0.247
Posterior	**F**_**(1,123)**_ **= 5.029, *p* = 0.008**	*t* = −1.575, *P*_FDR_ = 0.151	*t* = 1.600, *P*_FDR_ = 0.112
Whole thalamus	**F**_**(1,123)**_ **= 4.856, *p* = 0.009**	*t* = −1.726, *P*_FDR_ = 0.151	*t* = 1.388, *P*_FDR_ = 0.168

*Note:* The table reports results from MANCOVAs examining the ipsilateral subcortical structures (i.e., hippocampus, amygdala, and thalamus) and their sub‐segmentations. Significance levels are reported as *p*‐values adjusted for multiple comparisons using the false discovery rate (P_FDR_); P_FDR_ < 0.05 are highlighted in bold.

Abbreviations: AAA, anterior amygdaloid area; AB, accessory‐basal nuclei; BLA, basolateral amygdala (including lateral, basal, AB, and paralaminar nuclei); CAT, cortico‐amygdaloid transition area; CMA, central‐medial amygdala (including central and medial nuclei); HATA, hippocampal‐amygdaloid transition area.

Quantitative review of 3D‐FLAIR images of the hippocampi and amygdalae revealed no evidence of abnormal signal intensity, with no significant differences between clusters or in comparison with controls (Table S8).

Compared with TLE‐HS, Cluster 1 showed a widespread increase in cortical thickness, while Cluster 2 displayed regions of cortical atrophy (Figure 4B). Both clusters had larger ipsilateral hippocampus and amygdala volumes than TLE‐HS (Tables 2 and 3). Finally, direct comparison between the two TLE‐MRIneg clusters revealed global cortical atrophy in Cluster 2 relative to Cluster 1 (Figure S7), along with bilateral ventricular enlargement and reduced volumes in the contralateral basal ganglia and amygdala (Table S9).

#### 3.3.1 Latent Clinical Patterns Associated With Amygdala Increased Volume in Cluster 1

Pearson analyses revealed no significant correlations between AE and clinical variables (Table S10). Amygdala volume did not differ between drug‐resistant and drug‐responsive patients, nor between those with and without focal to bilateral tonic–clonic seizure (FBTCS; Table S11).

**FIGURE 5:**
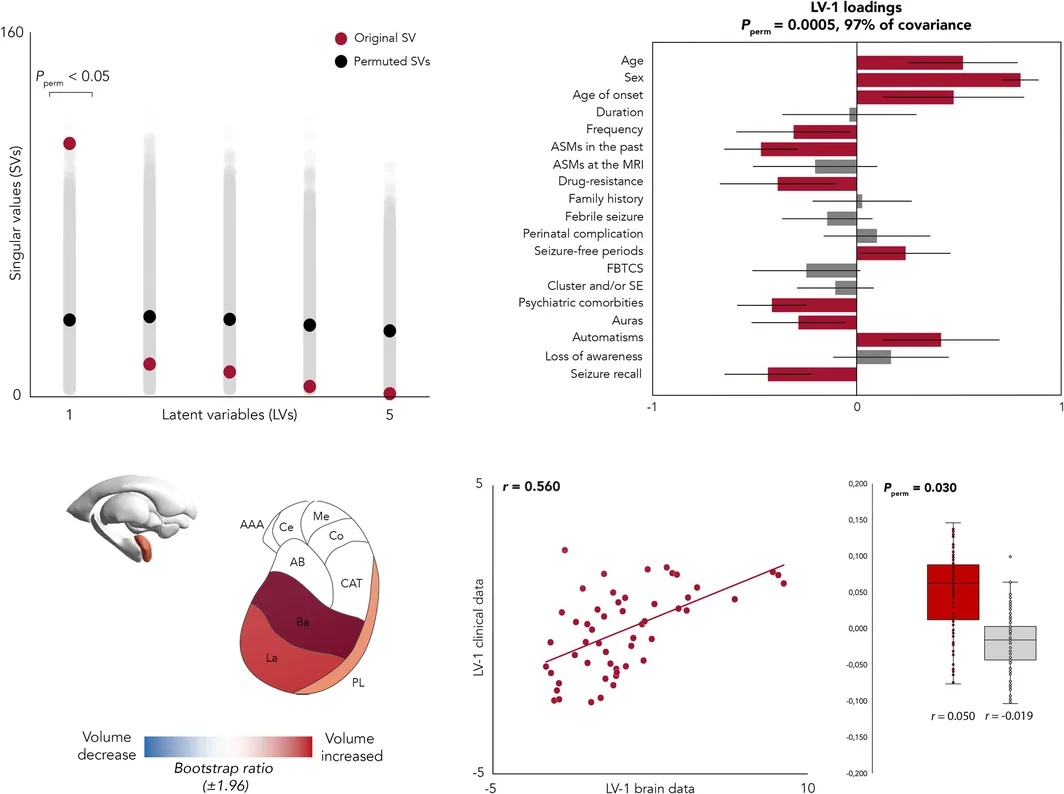
Multivariate association between clinical features and amygdala enlargement in TLE‐MRIneg Cluster 1. Partial least squares (PLS) analysis was used to examine associations between clinical variables and amygdala morphometry in patients with MRI‐negative temporal lobe epilepsy (TLE‐MRIneg), Cluster 1. The two datasets were correlated across patients and subjected to singular value decomposition, yielding latent variables (LVs, top left). Only LV‐1 explained significantly more covariance than expected under the null distribution (*p* < 0.05, top left), accounting for 97% of the total covariance (*P*_perm_ = 0.0005, top right). LV‐1 loadings indicate the contribution of each clinical variable. Red bars represent bootstrap ratios; error bars show bootstrap‐estimated standard errors (top right). Bootstrap ratios (±1.96) show the contribution of the amygdala and its subnuclei to LV‐1 (bottom left). Patients with increased amygdala volumes, especially in basolateral regions, were characterized by older age, later age at onset, lower seizure frequency and medication adjustments, more seizure‐free periods, fewer auras and psychiatric comorbidities, but greater automatisms and impaired seizure recall. Correlation between brain and clinical LV‐1 scores (*r* = 0.560) indicates a robust brain–behavior relationship (bottom center). Finally, cross‐validated out‐of‐sample correlation for held‐out data (*r* = 0.050) was greater than that observed in the permuted null model (*r* = −0.019, *P*_perm_ = 0.030, bottom right). ASMs: Antiseizure medications, FBTCS: Focal to bilateral tonic–clonic seizures, SE: Status epilepticus.

Despite the lack of significant univariate associations, PLS analysis identified one statistically significant latent variable (LV), which accounted for 97% of the covariance between clinical features and ipsilateral amygdala volume (*P*_perm_ = 0.0005, Figure 5). Within this cluster, the clinical profile was characterized by male gender, lower seizure frequency, fewer lifetime ASMs, positive response to treatment, absence of psychiatric comorbidities, older age at MRI, and later age of onset. In addition, fewer auras, more frequent automatisms, and lack of seizure recall in seizure semeiology appear to contribute to the observed AE morphometric profile. The correlation between individual patients' clinical profiles and amygdala volume was significant (*r* = 0.560), and out‐of‐sample cross‐validation confirmed that this association was unlikely to occur by chance (*P*_perm_ = 0.030).

#### 3.3.2 Latent Clinical Patterns Associated With Global Cortical Atrophy in Cluster 2

In Cluster 2, contralateral cortical thickness was mildly associated with seizure frequency (*r* = 0.404) and the number of ASMs (*r* = 0.392). No further linear correlations were found with other clinical variables, drug‐response, and/or FBTCS (Tables S10 and S11).

Although PLS analysis identified one LV accounting for 37% of the covariance (*P*_perm_ = 0.0004), cross‐validation did not support the robustness of this association. Full results are presented in Figure S8.

## 4 Discussion

This study builds on prior research by providing a comprehensive characterization of TLE with radiologically negative MRI. We identified non‐lesional TLE as a distinct clinical and morphological entity compared to TLE‐HS. Furthermore, we delineated two biologically meaningful subtypes within TLE‐MRIneg: one characterized by morphometric AE with unique neuroanatomical and clinical features, and another marked by widespread, symmetrical cortical atrophy.

### 4.1 Hippocampal Sclerosis and Non‐Lesional TLE: Two Sides of the Same Disease?

TLE is recognized as a progressive network disorder affecting both gray and white matter, linked to poor outcomes and cognitive comorbidities [54, 55, 56]. Structural abnormalities [18] and disrupted connectivity [57] are typically observed in conjunction with long disease duration, preceding precipitating events, poor seizure control, and drug resistance [58, 59, 60, 61]. Despite accounting for up to approximately 30% of TLE cases in previous reports [2], TLE‐MRIneg remains underexplored and diagnostically challenging. In the present study, conducted in a cohort of 209 TLE patients, 46% were classified as non‐lesional at the time of evaluation. This relatively high proportion of TLE‐MRIneg patients can be explained by a combination of imaging methodology and referral bias. All patients underwent brain MRI using epilepsy‐dedicated protocols compliant with HARNESS recommendations [28], contributing to greater consistency in the assessment of MRI findings across centers. In addition, our cohort was recruited exclusively from tertiary referral epilepsy centers, which are more likely to evaluate clinically complex and diagnostically challenging cases, including patients with inconclusive imaging findings. As a result, MRI‐negative cases are likely to be overrepresented in this setting compared with unselected or population‐based cohorts. Notably, many imaging studies have grouped TLE‐MRIneg patients with those affected by HS, potentially obscuring relevant biological and phenotypic differences between these subgroups [18, 19, 20, 21]. Recent artificial intelligence applications to structural MRI data have identified similar patterns of cortical atrophy in both MRI‐positive and MRI‐negative TLE, suggesting a continuum common to all TLE patients [13]. Differently, our findings support a reconceptualization of TLE‐MRIneg as a distinct clinical and neuroanatomical entity. At the time of imaging, TLE‐MRIneg patients exhibited a quite favorable clinical course in terms of seizure control, with nearly 75% achieving seizure freedom, fewer medication adjustments, and lower seizure frequency. They also had later epilepsy onset, no history of febrile seizures, no history of seizure clusters/SE, and more preserved awareness during seizures, consistent with prior reports [22]. Imaging revealed widespread cortical thinning and subcortical volume loss in TLE‐HS, whereas TLE‐MRIneg showed only minimal morphometric changes. These differences persisted after adjusting for age of onset and disease duration, indicating a possible distinct underlying biology. Additionally, when restricting the analyses to TLE‐MRIneg patients with clinical and neurophysiological evidence pointing to a mesial onset, the results confirmed the absence of cortical and subcortical mesial temporal abnormalities compared with healthy controls. However, the subdivision of the TLE‐MRIneg population into mesial and lateral subgroups remains speculative, as the lack of intracranial EEG and/or surgical outcome data in most of the patients prevents a definitive classification. Overall, our findings suggest that the diffuse atrophy commonly found in TLE [18, 19] may be largely driven by TLE‐HS patients. The structural profile of TLE‐MRIneg indicates lower disease burden and possibly different pathophysiology, highlighting the need to regard it as a separate clinical entity with implications for diagnosis, prognosis, and treatment management. A key strength of the present study lies in the depth of clinical phenotyping. This comprehensive clinical characterization may account for the emerging differences between recent evidence and earlier studies [18, 19, 20, 21], providing a more refined understanding of TLE‐MRIneg.

### 4.2 TLE‐MRIneg Phenotypes

Growing attention has been directed toward the heterogeneity within TLE. Data‐driven approaches have recently identified distinct neuroanatomical subtypes and disease trajectories [12, 62]. For instance, Jiang et al. [62] described four TLE subtypes with distinct structural, clinical, and treatment profiles: left and right HS, MRI‐negative TLE, and TLE with AE. Our findings are consistent with this classification. Specifically, we identified two morphometric phenotypes within the TLE‐MRIneg. Cluster 1 was characterized by preserved cortical thickness and subcortical volumes, alongside a focal increase in ipsilateral amygdala volume, predominantly within BLA (Figure 4). These findings are consistent with “Subtype 4” proposed by Jiang et al. [62], characterized by no cortical thickness or subcortical volume atrophy, but showing a significant increase in amygdala volume relative to both the healthy group and the other subtypes. PLS analysis identified specific clinical features associated with AE, including more favorable seizure control, later seizure onset, and older age (Figure 5), consistent with prior reports [8]. In contrast to earlier studies, we found no evidence linking AE to increased aura frequency or psychiatric symptoms [9, 10]. Importantly, none of the individuals in Cluster 1 met visual radiological criteria for AE. Indeed, 3D‐FLAIR imaging showed no abnormalities in amygdala signal intensity (Table S8), supporting the absence of visually undetected functional alterations. These findings contrast with prior reports of radiological AE, which is often accompanied by T2 and FLAIR signal abnormalities in up to 65% of cases [10]. We propose that these discrepancies may reflect either two distinct forms of AE, radiological versus morphometric, or a spectrum along a shared pathophysiological continuum. In our cohort, increased amygdala volume was present in 55% of TLE‐MRIneg cases, highlighting its prevalence and potential clinical relevance. In previous work, we reported that increased amygdala volume was associated with impairments in attention and processing speed [63]. More recently, we showed that AE was associated with ictal/postictal central apnea in TLE [64, 65]. Taken together, these findings support the hypothesis that morphometric AE may serve as a structural biomarker of a distinct disease phenotype. Further research is needed to elucidate its neurobiological basis and potential cognitive and psychiatric correlates.

As far as Cluster 2, it displays widespread bilateral cortical thinning, ventricular enlargement, and ipsilateral cortico‐medial amygdala atrophy. The cortical atrophy observed in Cluster 2 closely resembles “Subtype 3” described by Jiang and colleagues [62]. Although their patients were considerably younger, the imaging profiles show striking similarities. The authors termed this subtype “cortex‐predominant”, as patients exhibited gray matter loss mainly in the neocortex, with preserved subcortical volumes [62]. Moreover, no association was found between Cluster 2 cortical thickness and age and/or age of onset (Table S10). These results suggest that broad cortical atrophy in this subgroup may reflect a neuroanatomical feature, rather than a mere effect of aging. Interestingly, Cluster 2 amygdala changes resemble those described by our group in late‐onset TLE [39] patients, likely represented here. While whole amygdala volume remains stable with age [66], region‐specific atrophy has been reported [67], leaving unclear whether the observed cortico‐medial atrophy of amygdala reflects a disease‐specific marker of late‐onset TLE.

Taken together, these findings highlight distinct TLE‐MRIneg phenotypes, pointing to biological heterogeneity that extends beyond disease severity. Such morphometric heterogeneity may explain the absence of group‐level cortical thickness and subcortical amygdala differences between TLE‐HS and TLE‐MRIneg.

### 4.3 Limitations

We acknowledge study limitations. First, the relatively small sample size limited our ability to assess lateralized differences in TLE. To mitigate this, we used an ipsilateral/contralateral approach, as previously described [7, 19]. As a multicenter study, variability in MRI acquisition posed a potential bias, which we addressed through neuroCombat harmonization [36]. Harmonization was performed using the scanner as the batch variable, without the inclusion of biological covariates. This decision was deliberate and based on methodological considerations, in line with approaches adopted by large‐scale epilepsy neuroimaging initiatives [68]. However, all healthy controls were recruited from a single center, which represents an additional source of bias that could not be addressed through harmonization. Several aspects warrant further investigation. Future studies should incorporate multimodal imaging together with intracranial EEG and/or surgical outcome investigations to more comprehensively characterize phenotypes and epileptogenic networks in TLE‐MRIneg. Longitudinal data are also needed to determine whether non‐lesional TLE represents a distinct stable entity or, conversely, an early or milder variant of TLE associated with HS. Although our analyses did not reveal hippocampal alterations in TLE‐MRIneg patients, only longitudinal investigations could clarify whether the observed imaging‐negative phenotypes correspond to truly separate subgroups or reflect different stages along a continuum of disease. In addition, targeted neuropsychological assessments in patients with AE are essential to clarify potential cognitive impairments and inform individualized management strategies. Finally, the characterization of bitemporal cases was limited due to their low representation in our sample and should be more thoroughly examined in future work. Nevertheless, MRI‐negative bitemporal epilepsy did not appear to influence clustering, as bitemporal cases were equally distributed across both clusters.

## Author Contributions

Alice Ballerini: conceptualization, formal analysis, methodology, visualization, writing – original draft. Alessia Casarini: methodology, data curation, writing – review and editing. Niccolò Biagioli: data curation, writing – review and editing. Maurilio Genovese and Gaetano Cantalupo: methodology, writing – review and editing. Laura Mirandola, Daniela Ballotta, Paul Summers, Simona Scolastico, Laura Madrassi, Marcella Malagoli, Giada Giovannini, Matteo Pugnaghi, Niccolò Orlandi, Valeria Cuccarini, Domenico Aquino, Elena Tartara, and Fulvia Palesi: data collection, data curation, writing – review and editing. 3TLE Study Group: data collection. Laura Tassi, Giuseppe Didato, Paolo Vitali, and Stefano Meletti: conceptualization, writing – review and editing. Anna Elisabetta Vaudano: conceptualization, project administration, visualization, writing – original draft. 3TLE Study Group: Roberta Di Giacomo MD (Epilepsy Unit, Fondazione IRCCS Istituto Neurologico Carlo Besta, Milan, Italy), Fabio Doniselli MD (Neuroradiology Unit, Fondazione IRCCS Istituto Neurologico Carlo Besta, Milan, Italy), Federica Mazzi MD (Neuroradiology Unit, Fondazione IRCCS Istituto Neurologico Carlo Besta, Milan, Italy), Carlo Andrea Galimberti MD (Epilepsy Center, IRCCS Mondino Foundation, Pavia, Italy), and Claudia A.M. Gandini Wheeler‐Kingshott, PhD (Department of Brain and Behavioral Sciences, University of Pavia, Italy; Epilepsy Center, IRCCS Mondino Foundation, Pavia, Italy; NMR Research Unit, Department of Neuroinflammation, Queen Square Multiple Sclerosis Centre, UCL Queen Square Institute of Neurology, Faculty of Brain Sciences, UCL, London, UK).

## Funding

Data were collected within the 3TLE multicentric research project (Italian Ministry of Health, NET‐2013‐02355313).

## Conflicts of Interest

S.M. has received research grant support from the Italian Ministry of Health (MOH) and personal compensation as a member of the scientific advisory boards for UCB, Jazz Pharmaceuticals, and EISAI. A.E.V. has received speaker or consultancy fees from Angelini. All other authors declare no conflicts of interest.

## Supporting information


**Data S1:** acn370349‐sup‐0001‐DataS1.docx.

## Data Availability

The data that support the findings of this study are available from the corresponding author upon reasonable request.
