# Supplementary material for: Multidimensional Profiling of MRI‐Negative Temporal Lobe Epilepsy Uncovers Distinct Phenotypes
Source: Ann Clin Transl Neurol. 2026 Mar 24;13(9):1878–92. doi: 10.1002/acn3.70349 (PMC13394121; doi:10.1002/acn3.70349)
Supplement: Supplementary file 1 [file ACN3-13-1878-s001.docx]

**Supplementary Materials**

**Multidimensional profiling of MRI-negative Temporal Lobe Epilepsy**

**uncovers distinct phenotypes**

Alice Ballerini PhD, Alessia Casarini MSc, Niccolò Biagioli MD, Laura Mirandola MD PhD, Daniela Ballotta PhD, Paul Summers PhD, Simona Scolastico MD, Laura Madrassi MD, Maurilio Genovese MD, Marcella Malagoli MD, Gaetano Cantalupo MD, Giada Giovannini MD PhD, Matteo Pugnaghi MD PhD, Niccolò Orlandi MD, Laura Tassi MD, Valeria Cuccarini MD, Domenico Aquino MD, Elena Tartara MD, Fulvia Palesi PhD, Giuseppe Didato MD, Paolo Vitali MD PhD for the *3TLE Study Group*, Stefano Meletti MD PhD, Anna Elisabetta Vaudano MD PhD

**Supplementary Methods**

**Image acquisition and processing**

Three-dimensional structural MRI scans were acquired according to the HARNESS protocol guidelines^1^ using different 3T scanners across centers. Acquisition parameters for each sequence and center are summarized in **Supplementary Table 1**.

| **Center** | **Sequence** | **Instrumental Manufacture** | **Slice** | **Slice thickness (mm)** | **TR - TE (ms)** | **Flip Angle** | **Matrix** |
| --- | --- | --- | --- | --- | --- | --- | --- |
| 1 | 3D-T1w | Philips Ingenia | 208 | 1.1 | 7.4 - 3.5 | 8° | 220×210 |
| 1 | 3D-FLAIR | Philips Ingenia | 208 | 1.2 | 4800 - 294.2 | 90° | 200×200 |
| 1 | 3D-T1w | GE Signa Architect | 212 | 1.0 | 7.7 - 2182 | 8° | 256×256 |
| 1 | 3D-FLAIR | GE Signa Architect | 212 | 1.2 | 6002 - 117.7 | 90° | 256×230 |
| 2 | 3D-T1w | Siemens | 170 | 1.2 | 2300 - 2.95 | 9° | 256×240 |
| 2 | 3D-FLAIR | Siemens | 170 | 1.1 | 5000 - 388 | 120° | 320×288 |
| 3 | 3D-T1w | Philips Ingenia | 186 | 1.0 | 9.9 - 4.6 | 8° | 256×256 |
| 3 | 3D-FLAIR | Philips Ingenia | 186 | 1.0 | 4800 - 323.1 | 90° | 200×200 |

**Supplementary Table 1**. MRI sequence parameters across scanners and imaging centers. *Center 1: Baggiovara Civil Hospital (OCB; Modena, Italy); center 2: IRCCS Mondino Neurological Institute (Pavia, Italy); center 3: IRCCS Carlo Besta Neurological Institute (Milan, Italy). TR: repetition time, TE: echo time.*

***3D T1-weighted sequences***

Cortical thickness and subcortical volumes were obtained from the 3D T1-weighted (3D-T1) sequences using FreeSurfer v7.3.2 software^2^. A subject-specific cortical surface model was generated from each 3D-T1 scan, followed by cortical surface reconstruction and registration to the Conte69 template surface using Workbench tools^3–5^. The resulting cortical maps were spatially smoothed with a Gaussian kernel (full-width at half-maximum, FWHM=10 mm)^6^. Cortical thickness was computed as the distance between the white matter and pial surfaces, with measurements extracted from 32,000 vertices per hemisphere.

Subcortical segmentation was performed using FreeSurfer’s automated pipeline, yielding volumetric estimates for 16 subcortical structures. A dedicated FreeSurfer pipeline was also employed to subsegment the hippocampal subfields^7^, amygdala nuclei^8^, and thalamic nuclei^9^. The following hippocampal subfield volumes were extracted for each hemisphere: hippocampal body, hippocampal head, hippocampal tail, hippocampal fissure, subiculum, presubiculum, parasubiculum, CA1, CA2/3, CA4, molecular layer, granule cell and molecular layer of the dentate gyrus (GC-ML-DG), fimbria, and the hippocampal-amygdala transition area (HATA). Amygdala segmentation identified nine nuclei per hemisphere: anterior amygdaloid area (AAA), cortico-amygdaloid transition area (CAT), basal nucleus (Ba), lateral nucleus (La), accessory basal nucleus (AB), central nucleus (Ce), cortical nucleus (Co), medial nucleus (Me), and paralaminar nucleus (PL). As previously described by our group^10–13^, the amygdala subnuclei were clustered based on cytoarchitecture, histochemical properties, and connectivity patterns^14,15^ into three major complexes: *(i)* the basolateral amygdala (BLA) is the deepest complex, which includes La, Ba, AB and PL nuclei; *(ii)* the superficial group is named cortical amygdala (CA), and is composed by the Co nucleus only; *(iii)* finally, the central-medial amygdala (CMA) is composed of the Me and Ce nuclei. The thalamus was segmented into 25 nuclei per hemisphere, which were further clustered into six groups according to Iglesias and collaborators^9^: anterior, lateral, ventral, intralaminar, medial, and posterior groups. All segmentation and cortical reconstructions were subjected to a quality control procedure following standardized ENIGMA protocols (<http://enigma.usc.edu>).

***3D fluid-attenuated inversion recovery sequences***

Given the multicenter design and the heterogeneity in MRI scanners and 3D-FLAIR acquisition protocols (**Supplementary Table 1**), signal intensities were standardized before analysis. For each subject, 3D fluid-attenuated inversion recovery (3D-FLAIR) images of the TLE-MRIneg group only underwent skull stripping, bias-field correction using the N4 algorithm implemented in SimpleITK^16^, and rigid registration to the individual 3D-T1 image. Hippocampal and amygdalae regions of interest were defined using FreeSurfer-derived segmentations. Signal normalization was performed using a basal ganglion–based approach as previously described by Carré and colleagues^17^. Briefly, the mean and standard deviation of FLAIR signal were computed within bilateral basal ganglia masks and used to convert voxel intensities into subject-specific z-scores. The mean z-scored FLAIR signal was then extracted from the left and right hippocampus and amygdala for subsequent statistical analyses.

**Cluster analysis**

***Clinical phenotypes associated with brain morphometric features***

We first performed Pearson correlation analyses between epilepsy clinical variables and brain spatial patterns within each cluster to identify linear associations between clinical features and cortico-subcortical regions of interest (ROIs) that had been previously found to differ significantly from those of controls. Specifically, we assessed the relationships between age, age at epilepsy onset, disease duration, seizure frequency, and the number of ASMs, both at the time of MRI and historically, and the ROIs identified in case-control comparisons. In addition, within each cluster, patients were stratified based on drug-resistance status and history of FBTCS to examine their potential impact on brain morphology.

As far as brain morphometric data, in Cluster 1, we included the ipsilateral amygdala since it was the only subcortical ROI that resulted significantly different from controls. Thus, the following ipsilateral amygdalae ROIs were included in the analysis: La, Ba, PL, BLA, and whole amygdala. In Cluster 2, significant differences with controls were observed in the bilateral lateral ventricles, contralateral putamen, and ipsilateral amygdalae medial and cortical nuclei, as illustrated in **Figure 3** of the main text. Additionally, to reduce overfitting from the high-dimensional cortical data, the same down-sampling strategy applied during cluster analysis was used. Specifically, cortical surface data from Cluster 2 patients were processed using the ‘surface to parcel’ function from the ENIGMA-Toolbox^18^, reducing data to 180 ROIs per hemisphere according to the Glasser atlas^19^. A multivariate analysis of covariance (MANCOVA) was then performed between Cluster 2 and controls, using age and sex as covariates. ROIs with *P*-values that survived a 5% false discovery rate (FDR) correction^20^ were selected (**Supplementary Figure 1**). Pearson’s correlations on Cluster 2 were performed using the mean cortical thickness for both hemispheres.

**Supplementary Figure 1. Cluster 2 cortical regions that significantly differed from controls based on the Glasser atlas.** Only regions showing statistically significant effects after false discovery rate (FDR) correction (*P*_FDR_ < .05) are shown. All comparisons were adjusted for age, sex, and intracranial volume (ICV). Brain maps represent t-values and were generated using the ENIGMA Toolbox^18^.

Subsequently, partial least squares (PLS) analysis was performed separately within each cluster to determine clinical variable combinations that best explained the observed morphometric patterns. PLS is a multivariate, data-driven statistical method used to model linear combinations of two sets of variables that maximally covary with one another. PLS reduces data dimensionality by transforming the original variables into a new set of latent variables (LVs). These LVs aim to explain as much variance as possible in the response variables while being derived from the predictors. By reducing the number of components, PLS also addresses multicollinearity issues among predictor variables. The two sets of variables (i.e., brain data and clinical data) were correlated, and the resulting correlation matrix was subjected to singular value decomposition (SVD) using the “myPLS” toolbox for MATLAB^21,22^. SVD is the core PLS operation^23^ and extracts LVs that maximize the covariance between the two original datasets^24^.

We first prepared the dataset of clinical variables. The following clinical features were included in the PLS analysis: age, sex, family history, perinatal complications, febrile seizures, age at epilepsy onset, epilepsy duration, seizure frequency, number of ASMs at the time of MRI and in the past, drug resistance, seizure-free periods, FBTCS, seizure clusters and/or SE, psychiatric comorbidities, auras, automatisms, ictal responsiveness and impaired awareness during seizure recorde by VEEG, for both TLE-MRIneg clusters. Since PLS cannot handle missing data (NaNs), and some categorical clinical variables (such as family history, febrile seizures, perinatal complications, head trauma, seizure-free periods, FBTCS, seizure clusters and/or SE, psychiatric disorders, auras, and automatisms) included missing values, we applied a K-nearest neighbors (KNN) imputation (k=3). This method replaces NaNs by selecting the mode of the *k* nearest non-missing observations based on absolute distance. To reduce dimensionality and prevent overfitting, we merged several binary variables into aggregated scores. Specifically, the “auras” variable included epigastric, auditory, visual, olfactory, gustatory, autonomic, psychic, and sensory-motor auras (both unilateral and bilateral), with a resulting score from 0 to 9 based on the number of aura types reported. Similarly, “automatisms” included oral, manual (unilateral and bilateral), and hyperkinetic automatisms, resulting in a score from 0 to 4.

**Supplementary Results**

**Supplementary Figure 2. Individual z-score analyses of hippocampal subfield volumes and FLAIR signal intensity in TLE-MRIneg patients.** Volumetric z-scores (black dots) are shown for the hippocampal head, body, and tail; hippocampal 3D-FLAIR signal intensity z-scores are shown in pink. Each dot represents an individual TLE-MRIneg patient. Dashed lines indicate −2 standard deviations (SD) and +1.5 SD thresholds relative to controls.

| *a)* | **TLE** | | **Controls** | **Stat** | **P-value** | |
| --- | --- | --- | --- | --- | --- | --- |
| N | 172 | | 102 |  |  | |
| Age (y) | 42.16(±14.872) | | 40.43(±16.576) | 0.889^t^ | 0.375 | |
| Sex (f/m) | 98/74 | | 57/45 | 0.031^χ²^ | 0.860 | |
| ICV (mm^3^) | 1×10^6^(±2×10^5^) | | 2×10^6^(±2×10^5^) | 3.513^t^ | **<0.001** | |
| *b)* | **TLE-MRIneg** | **TLE-HS** | **Controls** | **Stat** | **P-value** | **Pairwise comparisons** |
| N | 96 | 76 | 102 |  |  |  |
| Age (y) | 43.43(±16.967) | 40.55(±11.615) | 40.43(±16.576) | 1.124^F^ | 0.326 |  |
| Sex (f/m) | 55/41 | 43/33 | 57/45 | 0.040^χ²^ | 0.980 |  |
| ICV (mm^3^) | 1×10^6^(±1×10^5^) | 1×10^6^(±2×10^5^) | 2×10^6^(±2×10^5^) | 6.168^F^ | **0.002** | TLE-HS (P_FDR_=0.006)  TLE-MRIneg (P_FDR_=0.004) |

**Supplementary Table 2**. Demographic comparisons between TLE and controls. *Age and intracranial volumes (ICV) are presented as mean (± standard deviation).* *Age is expressed in years (y) at the time of the MRI scan, and ICV is expressed in mm^3^. Sex is represented as “female” (f) and “male” (m). t: independent sample t-test, χ²: chi-square test, F: ANOVA, P_FDR_: P-values adjusted using FDR correction. Portion a) shows the demographic comparisons between TLE and controls, while portion b) shows the comparison between TLE-MRIneg, TLE-HS, and controls. The pairwise comparison column shows the comparison between TLE subgroups and controls.*

|  | **Left side** | **Right side** | **Paired t-test** | **P-value** |
| --- | --- | --- | --- | --- |
| Hippocampal tail | 0.194(±0.894) | 0.204(±0.819) | -0.177 | 0.859 |
| Hippocampal body | 0.211(±0.851) | 0.249(±0.791) | -0.665 | 0.508 |
| Hippocampal head | 0.221(±0.840) | 0.238(±0.726) | -0.339 | 0.735 |
| Whole hippocampus | 0.245(±0.821) | 0.263(±0.715) | -0.410 | 0.683 |
| Whole amygdala | 0.188(±0.857) | 0.227(±0.746) | -0.857 | 0.393 |

**Supplementary Table 3**. Hippocampus and amygdala symmetry analysis in healthy controls. *Data are presented in z-score as mean (± standard deviation). Paired-sample t-tests were conducted on healthy controls (N=102) to assess potential asymmetries between the left and right hippocampi and amygdalae.*

| **ROI** | | **Controls vs TLE-HS** | | **Controls vs TLE-MRIneg** | | **TLE-MRIneg vs TLE-HS** | |
| --- | --- | --- | --- | --- | --- | --- | --- |
|  |  | **Stat (t)** | **Sign. (P_FDR_)** | **Stat (t)** | **Sign. (P_FDR_)** | **Stat (t)** | **Sign. (P_FDR_)** |
| Ipsilateral lateral ventricle | | -3.515 | **0.001** | -2.929 | **0.037** | -0.749 | 0.455 |
| Contralateral lateral ventricle | | -3.157 | **0.002** | -2.508 | 0.064 | -0.788 | 0.431 |
| Ipsilateral hippocampus | |  |  |  |  |  |  |
|  | Subiculum | 9.079 | **<0.001** | -0.464 | 0.643 | 9.540 | **<0.001** |
|  | Presubiculum | 9.026 | **<0.001** | -0.713 | 0.477 | 9.731 | **<0.001** |
|  | Parasubiculum | 4.026 | **<0.001** | -1.153 | 0.250 | 5.133 | **<0.001** |
|  | CA1 | 10.016 | **<0.001** | 0.515 | 0.607 | 9.555 | **<0.001** |
|  | CA3 | 7.723 | **<0.001** | -0.261 | 0.794 | 7.994 | **<0.001** |
|  | CA4 | 10.659 | **<0.001** | 0.128 | 0.898 | 10.570 | **<0.001** |
|  | Dental gyrus | 10.497 | **<0.001** | 0.123 | 0.903 | 10.416 | **<0.001** |
|  | Molecular layer | 10.943 | **<0.001** | 0.026 | 0.979 | 10.950 | **<0.001** |
|  | Hippocampal fissure | 1.280 | 0.202 | 1.349 | 0.178 | 0.005 | 0.996 |
|  | Fimbria | 3.499 | **0.001** | -0.014 | 0.988 | 3.526 | **<0.001** |
|  | HATA | 2.739 | **0.007** | -0.189 | 0.850 | 2.927 | **0.004** |
|  | Body | 10.763 | **<0.001** | -0.288 | 0.773 | 11.071 | **<0.001** |
|  | Head | 9.869 | **<0.001** | 0.171 | 0.865 | 9.736 | **<0.001** |
|  | Tail | 8.186 | **<0.001** | 0.396 | 0.693 | 7.836 | **<0.001** |
|  | Whole hippocampus | 10.922 | **<0.001** | 0.150 | 0.881 | 10.812 | **<0.001** |
| Ipsilateral amygdala | |  |  |  |  |  |  |
|  | Lateral nucleus | 3.386 | **0.001** | -2.655 | 0.101 | 5.913 | **<0.001** |
|  | Basal nucleus | 3.764 | **<0.001** | -2.130 | 0.147 | 5.794 | **<0.001** |
|  | AB nucleus | 3.350 | **0.001** | -0.571 | 0.615 | 3.900 | **<0.001** |
|  | AAA | 3.486 | **0.001** | -1.829 | 0.147 | 5.228 | **<0.001** |
|  | Central nucleus | 3.783 | **<0.001** | -0.503 | 0.615 | 4.270 | **<0.001** |
|  | Medial nucleus | 3.546 | **0.001** | 1.696 | 0.156 | 1.949 | 0.052 |
|  | Cortical nucleus | 3.835 | **<0.001** | 1.339 | 0.272 | 2.579 | **0.012** |
|  | CAT | 3.174 | **0.002** | -0.940 | 0.464 | 4.076 | **<0.001** |
|  | Paralaminar nucleus | 4.393 | **<0.001** | -1.957 | 0.147 | 6.262 | **<0.001** |
|  | BLA | 3.994 | **<0.001** | -1.921 | 0.147 | 5.824 | **<0.001** |
|  | CMA | 4.046 | **<0.001** | 0.665 | 0.608 | 3.431 | **0.001** |
|  | Whole amygdala | 3.897 | **<0.001** | -1.796 | 0.147 | 5.612 | **<0.001** |
| Ipsilateral thalamus | |  |  |  |  |  |  |
|  | Anterior | 2.999 | **0.003** | 0.192 | 0.848 | 2.827 | **0.011** |
|  | Lateral | 2.761 | **0.006** | -0.011 | 0.991 | 2.778 | **0.011** |
|  | Ventral | 1.133 | 0.258 | -1.287 | 0.199 | 2.360 | **0.022** |
|  | Intralaminar | 1.014 | 0.311 | -0.961 | 0.338 | 1.928 | 0.055 |
|  | Medial | 2.979 | **0.003** | -0.180 | 0.857 | 3.159 | **0.007** |
|  | Posterior | 2.175 | **0.030** | -0.471 | 0.638 | 2.629 | **0.013** |
|  | Whole thalamus | 2.585 | **0.010** | -0.644 | 0.520 | 3.206 | **0.007** |

**Supplementary Table 4**. Subcortical volume comparisons between TLE-HS, TLE-MRIneg, and controls. *The table reports results from pairwise MANCOVA comparisons examining bilateral lateral ventricles, ipsilateral subcortical structures, and their sub-segmentations. “t” indicates the t-value from each comparison (i.e., controls vs TLE-HS, controls vs TLE-MRIneg, or TLE-MRIneg and TLE-HS); significance levels are reported as P-values adjusted for multiple comparisons using the false discovery rate (P_FDR_). Significant results are highlighted in BOLD. HATA: hippocampal-amygdaloid transition area, AB: accessory-basal nuclei, AAA: anterior amygdaloid area, CAT: cortico-amygdaloid transition area, BLA: basolateral amygdala (including lateral, basal, AB, and paralaminar nuclei), CMA: central-medial amygdala (including central and medial nuclei).*

**Supplementary Figure 3. Whole-brain comparisons between TLE patients and healthy controls.** The figure illustrates the unthresholded group differences t-values of cortical thickness and subcortical volumes maps between healthy controls and patients with temporal lobe epilepsy (TLE). Panel A depicts the comparison with the entire TLE cohort, panel B shows patients with hippocampal sclerosis (TLE-HS), and panel C shows patients with MRI-negative TLE (TLE-MRIneg). Finally, panel D presents the direct comparison between the two TLE subgroups (TLE-MRIneg vs TLE-HS). All comparisons were adjusted for age and sex, and subcortical comparisons were additionally adjusted for intracranial volume (ICV). Brain maps represent t-values and were generated using the ENIGMA Toolbox^18^.

|  | **TLE-MRIneg** | **TLE-HS** | **Stat.** | **Sign.** |
| --- | --- | --- | --- | --- |
| N | 42 | 42 |  |  |
| Age | 37.19(±11.543) | 37.07(±12.611) | 0.002^t^ | 0.964 |
| Sex (f/m) | 23/19 | 27/15 | 0.791^χ²^ | 0.374 |
| Age of onset | 25.87(±14.097) | 25.45(±15.345) | 0.017^t^ | 0.897 |
| Duration | 11.62(±7.516) | 11.64(±7.515) | 0.000^t^ | 0.988 |
| Hemisphere |  |  | 0.963^χ²^ | 0.618 |
| *Left temporal lobe* | 29 | 25 |  |  |
| *Right temporal lobe* | 12 | 15 |  |  |
| *Bitemporal* | 1 | 2 |  |  |
| Frequency | 3.05(±1.513) | 3.79(±1.094) | 6.561^t^ | **0.012** |
| Number of ASMs at the MRI | 1.55(±0.832) | 2.00(±0.796) | 6.477^t^ | **0.013** |
| Number of ASMs in the past | 0.95(±1.561) | 1.09(±1.185) | 0.223^t^ | 0.638 |
| Drug-resistance (y/n) | 14/28 | 16/26 | 0.207^χ²^ | 0.649 |
| Family history (y/n)^a^ | 10/31 | 8/34 | 0.349^χ²^ | 0.555 |
| Febrile seizure (y/n)^b^ | 3/39 | 16/24 | 12.425^χ²^ | **<0.001** |
| Perinatal complications (y/n) | 2/40 | 2/40 | 0.000^χ²^ | 0.999 |
| Head trauma (y/n) | 2/40 | 4/38 | 0.718^χ²^ | 0.397 |
| Seizure-free periods (y/n) | 17/25 | 13/29 | 0.830^χ²^ | 0.362 |
| FBTCS (y/n) | 28/14 | 29/13 | 0.055^χ²^ | 0.815 |
| Cluster and/or SE (y/n) | 10/32 | 15/27 | 1.424^χ²^ | 0.233 |
| Falls related to epilepsy (y/n)^a^ | 6/35 | 13/29 | 3.130^χ²^ | 0.077 |
| PNES (y/n) | 0/42 | 0/42 | - | - |
| Psychiatric disorders (y/n) |  |  | 5.083^χ²^ | 0.079 |
| *None* | 26 | 35 |  |  |
| *Mixed anxiety-depression* | 13 | 5 |  |  |
| *Psychosis* | 3 | 2 |  |  |
| Neurosurgery (y/n) | 0/42 | 21/21 | 28.000^χ²^ | **<0.001** |
| Slow-wave activity (y/n) | 31/11 | 28/14 | 0.513^χ²^ | 0.474 |
| Epileptiform activity (y/n) | 37/5 | 36/6 | 0.105^χ²^ | 0.746 |
| Loss of awareness (y/n)^c^ | 21/20 | 27/12 | 2.702^χ²^ | 0.100 |
| Seizure recall (y/n)^d^ | 15/23 | 10/28 | 1.490^χ²^ | 0.222 |
| Aphasia (y/n)^e^ | 18/20 | 17/21 | 0.053^χ²^ | 0.818 |
| Confusion (y/n)^d^ | 22/16 | 20/18 | 0.213^χ²^ | 0.645 |
| Epigastric aura (y/n)^b^ | 12/30 | 21/19 | 4.878^χ²^ | **0.027** |
| Auditory aura (y/n)^b^ | 2/40 | 2/38 | 0.003^χ²^ | 0.960 |
| Visual aura (y/n)^b^ | 4/38 | 3/37 | 0.107^χ²^ | 0.743 |
| Olfactory aura (y/n)^b^ | 2/40 | 0/40 | 1.952^χ²^ | 0.162 |
| Gustatory aura (y/n)^b^ | 1/41 | 4/36 | 2.077^χ²^ | 0.150 |
| Autonomic aura (y/n)^b^ | 18/24 | 15/25 | 0.245^χ²^ | 0.621 |
| Psychic aura (y/n)^b^ | 22/20 | 22/18 | 0.057^χ²^ | 0.812 |
| Unilateral sensory-motor aura (y/n)^b^ | 2/40 | 2/38 | 0.003^χ²^ | 0.960 |
| Bilateral sensory-motor aura (y/n)^b^ | 1/41 | 1/39 | 0.001^χ²^ | 0.972 |
| Oral automatism (y/n)^f^ | 7/32 | 22/17 | 12.350^χ²^ | **<0.001** |
| Unilateral manual automatism (y/n)^e^ | 3/37 | 5/34 | 0.614^χ²^ | 0.433 |
| Bilateral manual automatism (y/n)^f^ | 3/36 | 9/30 | 3.545^χ²^ | 0.060 |
| Hyperkinetic automatism (y/n)^e^ | 2/38 | 0/39 | 2.001^χ²^ | 0.157 |

**Supplementary Table 5**. Clinical comparison between TLE-MRIneg and TLE-HS selected by post hoc 1:1 nearest neighbor matching analysis. *Age, age of onset, epilepsy duration, frequency, and antiseizure medication (ASM) are presented as mean (± standard deviation). Age, age of onset, and epilepsy duration are expressed as years (y). The seizure frequency is described as follows: (1) less than one seizure per year, (2) one to three seizures per year, (3) four to eleven seizures per year, (4) one to three seizures per month, (5) one to six seizures per week, (6) one to three seizures per day, and (7) more than four seizures per day. The other variables are expressed as "yes" (y) and "no" (n), and sex is represented as "female" (f) and "male" (m). None of the included patients showed psychogenic non-epileptic seizure (PNES). Significant results are highlighted in BOLD. t: independent-sample t-test, χ²: chi-squared test, FBTCS: focal to bilateral tonic-clonic seizure, SE: status epilepticus. The level of consciousness is defined by ictal loss of awareness and seizure recall. Data are missing for the following patients: ^a^1, ^b^2, ^c^4, ^d^8, ^e^5, and ^f^6.*

**Supplementary Figure 4. Whole-brain comparisons between mesial TLE-MRIneg patients, healthy controls, and TLE-HS.** Group differences in cortical thickness and subcortical volumes between patients with mesial TLE-MRIneg (N=79) and healthy controls (**A**), and between mesial TLE-MRIneg and TLE-HS (**B**). Only regions showing statistically significant effects after false discovery rate (FDR) correction (*P*_FDR_ < 0.05) are shown. All comparisons were adjusted for age and sex, and subcortical comparisons were additionally adjusted for intracranial volume (ICV). Brain maps represent t-values and were generated using the ENIGMA Toolbox^18^.

| **ROI** | | **Controls vs. mesial TLE-MRIneg** | | **TLE-HS vs. mesial TLE-MRIneg** | |
| --- | --- | --- | --- | --- | --- |
|  |  | **Stat (t)** | **Sign. (P_FDR_)** | **Stat (t)** | **Sign. (P_FDR_)** |
| Ipsilateral hippocampus | |  |  |  |  |
|  | Subiculum | -1.079 | 0.281 | -9.525 | **<0.001** |
|  | Presubiculum | -1.469 | 0.143 | -9.975 | **<0.001** |
|  | Parasubiculum | -1.565 | 0.119 | -5.319 | **<0.001** |
|  | CA1 | -0.073 | 0.942 | -9.520 | **<0.001** |
|  | CA3 | -0.789 | 0.431 | -7.966 | **<0.001** |
|  | CA4 | -0.491 | 0.624 | -10.443 | **<0.001** |
|  | Dental gyrus | -0.559 | 0.577 | -10.344 | **<0.001** |
|  | Molecular layer | -0.723 | 0.470 | -10.967 | **<0.001** |
|  | Hippocampal fissure | 1.228 | 0.220 | 0.035 | 0.972 |
|  | Fimbria | -0.417 | 0.677 | -3.699 | **<0.001** |
|  | HATA | -0.643 | 0.521 | -3.192 | **0.002** |
|  | Body | -1.034 | 0.302 | -11.052 | **<0.001** |
|  | Head | -0.502 | 0.616 | -9.855 | **<0.001** |
|  | Tail | -0.453 | 0.651 | -8.143 | **<0.001** |
|  | Whole hippocampus | -0.681 | 0.496 | -10.949 | **<0.001** |
| Ipsilateral amygdala | |  |  |  |  |
|  | Lateral nucleus | -2.607 | 0.101 | -5.606 | **<0.001** |
|  | Basal nucleus | -2.330 | 0.101 | -5.739 | **<0.001** |
|  | AB nucleus | -1.043 | 0.397 | -4.150 | **<0.001** |
|  | AAA | -1.964 | 0.101 | -5.141 | **<0.001** |
|  | Central nucleus | -0.824 | 0.448 | -4.405 | **<0.001** |
|  | Medial nucleus | 1.759 | 0.137 | -1.733 | 0.084 |
|  | Cortical nucleus | 0.912 | 0.435 | -2.763 | **0.007** |
|  | CAT | -1.393 | 0.247 | -4.311 | **<0.001** |
|  | Paralaminar nucleus | -2.004 | 0.101 | -6.030 | **<0.001** |
|  | BLA | -2.118 | 0.101 | -5.763 | **<0.001** |
|  | CMA | 0.521 | 0.603 | -3.384 | **0.001** |
|  | Whole amygdala | -2.009 | 0.101 | -5.568 | **<0.001** |
| Ipsilateral thalamus | |  |  |  |  |
|  | Anterior | -0.059 | 0.953 | -2.963 | **0.008** |
|  | Lateral | -0.191 | 0.849 | -2.851 | **0.008** |
|  | Ventral | -1.439 | 0.151 | -2.509 | **0.015** |
|  | Intralaminar | -1.365 | 0.174 | -2.336 | **0.020** |
|  | Medial | -0.314 | 0.754 | -3.153 | **0.006** |
|  | Posterior | -0.795 | 0.428 | -2.784 | **0.008** |
|  | Whole thalamus | -0.931 | 0.353 | -3.347 | **0.006** |

**Supplementary Table 6**. Subcortical volume comparisons between mesial TLE-MRIneg, controls, and TLE-HS. *The table reports results from pairwise MANCOVA comparisons examining bilateral lateral ventricles, ipsilateral subcortical structures, and their sub-segmentations. “t” indicates the t-value from each comparison (i.e., controls and mesial TLE-MRIneg or TLE-HS and mesial TLE-MRIneg); significance levels are reported as P-values adjusted for multiple comparisons using the false discovery rate (P_FDR_). Significant results are highlighted in BOLD. HATA: hippocampal-amygdaloid transition area, AB: accessory-basal nuclei, AAA: anterior amygdaloid area, CAT: cortico-amygdaloid transition area, BLA: basolateral amygdala (including lateral, basal, AB, and paralaminar nuclei), CMA: central-medial amygdala (including central and medial nuclei).*

**Supplementary Figure 5. Clustering procedure and dimensionality reduction in TLE-MRIneg.** (**A**) The “Elbow method” is used to determine the optimal number of clusters, based on the reduction in within-cluster sum of squared errors (SSE). A marked inflection point at *k*=2 supports a two-cluster solution. (**B**) Principal component analysis (PCA) of the clustering solution, displaying individual participants in the space of the first two components. Patients are color-coded by cluster membership, showing a clear separation between the two subgroups.

|  | **TLE-MRIneg**  **Cluster 1** | **TLE-MRIneg**  **Cluster 2** | **TLE-HS** | **Stat.** | **Sign.** | **Pairwise** |
| --- | --- | --- | --- | --- | --- | --- |
| N | 53 | 43 | 76 |  |  |  |
| Age | 38.42  (±15.739) | 49.60  (±15.646) | 40.55  (±11.615) | 8.139^F^ | **<0.001** | C2 > C1 (P_FDR_<0.001)  C2 > HS (P_FDR_=0.001) |
| Sex (f/m) | 26/27 | 29/14 | 43/33 | 3.282^χ²^ | 0.194 |  |
| Age of onset | 29.58  (±17.996) | 39.88  (±22.559) | 18.72  (±14.416) | 19.904^F^ | **<0.001** | HS < C1 (P_FDR_=0.001)  HS < C2 (P_FDR_<0.001)  C1 < C2 (P_FDR_=0.005) |
| Duration | 8.90  (±10.212) | 10.14  (±11.825) | 21.72  (±13.710) | 21.375^F^ | **<0.001** | HS > C1 (P_FDR_<0.001)  HS > C2 (P_FDR_<0.001) |
| Hemisphere |  |  |  | 7.672^χ²^ | 0.104 |  |
| *Left temporal lobe* | 33 | 30 | 45 |  |  |  |
| *Right temporal lobe* | 17 | 8 | 29 |  |  |  |
| *Bitemporal* | 3 | 5 | 2 |  |  |  |
| Mesial (y/n) | 43/10 | 36/7 | 76/0 | 15.113^χ²^ | **<0.001** | HS > C1 (P_FDR_<0.001)  HS > C2 (P_FDR_<0.001) |
| Frequency | 2.91  (±1.348) | 3.02  (±1.439) | 3.85  (±1.186) | 10.147^F^ | **<0.001** | HS > C1 (P_FDR_<0.001)  HS > C2 (P_FDR_=0.001) |
| Number of ASMs at the MRI | 1.55  (±0.774) | 1.65  (±0.973) | 2.23  (±0.879) | 11.233^F^ | **<0.001** | HS > C1 (P_FDR_<0.001)  HS > C2 (P_FDR_=0.001) |
| Number of ASMs in the past | 0.7  4(±1.303) | 1.05  (±2.171) | 1.69  (±1.442) | 5.818^F^ | **0.004** | HS > C1 (P_FDR_=0.003) |
| Drug-resistance (y/n) | 12/41 | 12/31 | 43/33 | 18.065^χ²^ | **<0.001** | HS > C1 (P_FDR_<0.001)  HS > C2 (P_FDR_=0.004) |
| Family history (y/n)^a^ | 12/38 | 6/36 | 13/61 | 1.631^χ²^ | 0.443 |  |
| Febrile seizure (y/n)^b^ | 5/48 | 1/42 | 31/42 | 32.507^χ²^ | **<0.001** | HS > C1 (P_FDR_<0.001)  HS > C2 (P_FDR_<0.001) |
| Perinatal complications (y/n)^c^ | 5/48 | 2/41 | 8/67 | 1.278^χ²^ | 0.528 |  |
| Head trauma (y/n)^c^ | 6/47 | 2/41 | 8/67 | 1.515^χ²^ | 0.469 |  |
| Seizure-free periods (y/n)^c^ | 19/34 | 12/31 | 26/49 | .781^χ²^ | 0.677 |  |
| FBTCS (y/n)^c^ | 35/18 | 23/20 | 49/26 | 2.031^χ²^ | 0.362 |  |
| Cluster and/or SE (y/n)^c^ | 9/44 | 10/33 | 30/46 | 8.525^χ²^ | **0.014** | HS > C1 (P_FDR_=0.018) |
| Falls related to epilepsy (y/n)^d^ | 5/47 | 7/36 | 28/47 | 14.794^χ²^ | **0.001** | HS > C1 (P_FDR_<0.001)  HS > C2 (P_FDR_=0.024) |
| PNES (y/n)^c^ | 0/53 | 0/43 | 3/72 | 3.90 ^χ²^ | 0.142 |  |
| Psychiatric disorders (y/n)^c^ |  |  |  | 4.393^χ²^ | 0.355 |  |
| *None* | 39 | 30 | 63 |  |  |  |
| *Mixed anxiety-depression* | 13 | 11 | 10 |  |  |  |
| *Psychosis* | 1 | 2 | 2 |  |  |  |
| Neurosurgery (y/n) | 2/51 | 2/41 | 39/37 | 50.302^χ²^ | **<0.001** | HS > C1 (P_FDR_<0.001)  HS > C2 (P_FDR_<0.001) |
| Slow-wave activity (y/n) | 40/13 | 31/12 | 57/19 | .167^χ²^ | 0.920 |  |
| Epileptiform activity (y/n) | 44/9 | 33/10 | 64/12 | 1.092^χ²^ | 0.579 |  |
| Loss of awareness (y/n)^a^ | 24/28 | 26/16 | 54/19 | 10.007^χ²^ | **0.007** | HS > C1 (P_FDR_=0.006) |
| Seizure recall (y/n)^e^ | 22/27 | 11/29 | 20/51 | 4.424^χ²^ | 0.109 |  |
| Aphasia (y/n)^f^ | 21/29 | 19/22 | 31/39 | .174^χ²^ | 0.917 |  |
| Confusion (y/n)^e^ | 26/24 | 24/17 | 40/29 | .537^χ²^ | 0.764 |  |
| Epigastric aura (y/n)^b^ | 16/37 | 15/27 | 38/36 | 6.330^χ²^ | **0.042** |  |
| Auditory aura (y/n)^b^ | 4/49 | 4/38 | 3/71 | 1.454^χ²^ | 0.483 |  |
| Visual aura (y/n)^b^ | 7/46 | 2/40 | 3/71 | 4.386^χ²^ | 0.112 |  |
| Olfactory aura (y/n)^b^ | 3/50 | 2/40 | 1/73 | 1.914^χ²^ | 0.384 |  |
| Gustatory aura (y/n)^b^ | 2/51 | 0/42 | 4/70 | 2.297^χ²^ | 0.317 |  |
| Autonomic aura (y/n)^b^ | 23/30 | 19/23 | 22/52 | 3.741^χ²^ | 0.154 |  |
| Psychic aura (y/n)^b^ | 24/29 | 14/28 | 34/40 | 1.970^χ²^ | 0.373 |  |
| Unilateral sensory-motor aura (y/n)^b^ | 2/51 | 2/40 | 4/70 | .182^χ²^ | 0.913 |  |
| Bilateral sensory-motor aura (y/n)^b^ | 1/52 | 0/42 | 4/70 | 3.036^χ²^ | 0.219 |  |
| Oral automatism (y/n)^g^ | 14/36 | 9/32 | 41/32 | 16.589^χ²^ | **<0.001** | HS > C1 (P_FDR_=0.003)  HS > C2 (P_FDR_<0.001) |
| Unilateral manual automatism (y/n)^h^ | 9/42 | 3/38 | 15/58 | 3.447^χ²^ | 0.178 |  |
| Bilateral manual automatism (y/n)^g^ | 8/42 | 2/39 | 13/60 | 3.873^χ²^ | 0.144 |  |
| Hyperkinetic automatism (y/n)^g^ | 4/46 | 1/40 | 1/72 | 3.932^χ²^ | 0.140 |  |

**Supplementary Table 7**. Clinical comparison between TLE-MRIneg clusters and TLE-HS. *Age, age of onset, epilepsy duration, frequency, and antiseizure medication (ASM) are presented as mean (± standard deviation). Age, age of onset, and epilepsy duration are expressed as years (y). The seizure frequency is described as follows: (1) less than one seizure per year, (2) one to three seizures per year, (3) four to eleven seizures per year, (4) one to three seizures per month, (5) one to six seizures per week, (6) one to three seizures per day, and (7) more than four seizures per day. The other variables are expressed as "yes" (y) and "no" (n), and sex is represented as "female" (f) and "male" (m). Significant results are highlighted in BOLD. F: ANOVA, χ²: chi-squared test, FBTCS: focal to bilateral tonic-clonic seizure, SE: status epilepticus, PNES: psychogenic non-epileptic seizure. The level of consciousness is defined by ictal loss of awareness and seizure recall. Data are missing for the following patients: ^a^5, ^b^3, ^c^1, ^d^2, ^e^12, ^f^11, ^g^8, and ^h^7.*

**Supplementary Figure 6.** **Cortico-subcortical patterns across TLE-MRIneg clusters.** The figure illustrates whole-brain comparisons between MRI-negative temporal lobe epilepsy (TLE-MRIneg) Cluster 1 and Cluster 2, and both healthy controls and patients with hippocampal sclerosis (TLE-HS). Panel **A** shows differences between healthy controls and TLE-MRIneg Cluster 1 (**A1**) and Cluster 2 (**A2**). Panel **B** depicts differences between TLE-HS and TLE-MRIneg Cluster 1 (**B1**) and Cluster 2 (**B2**). All comparisons were adjusted for age and sex, and subcortical comparisons were additionally adjusted for intracranial volume (ICV). Brain maps represent unthresholded t-values and were generated using the ENIGMA Toolbox^18^.

| **ROI** | **TLE-MRIneg Cluster 1** | **TLE-MRIneg Cluster 2** | **Controls** | **Stat.** | ***P*-value** |
| --- | --- | --- | --- | --- | --- |
| Ipsilateral amygdala | 1.37(±0.252) | 1.38(±0.368) | 1.41 (±0.265) | 0.330^F^ | 0.720 |
| Ipsilateral hippocampus | 1.13(±0.279) | 1.20(±0.335) | 1.22(±0.249) | 1.167^F^ | 0.314 |
| Contralateral amygdala | 1.34(±0.276) | 1.38(±0.299) | 1.45(±0.266) | 1.758^F^ | 0.176 |
| Contralateral hippocampus | 1.11(±0.263) | 1.20(±0.340) | 1.25(±0.246) | 2.326^F^ | 0.102 |

**Supplementary Table 8**. Comparison of FLAIR signal intensity between TLE-MRIneg clusters and controls. *Values represent ANCOVA F-statistics comparing Cluster 1, Cluster 2, and controls, with age and sex included as covariates. ROI: region of interest.*

**Supplementary Figure 7. Whole-brain comparisons between TLE-MRIneg Cluster 2 versus Cluster 1.** Group differences in cortical thickness and subcortical volumes between patients with TLE-MRIneg Cluster 2 (N=43) and Clsuter 1 (N=53). Only regions showing statistically significant effects after false discovery rate (FDR) correction (*P*_FDR_ < 0.05) are shown. All comparisons were adjusted for age and sex, and subcortical comparisons were additionally adjusted for intracranial volume (ICV). Brain maps represent t-values and were generated using the ENIGMA Toolbox^18^.

| **TLE-MRIneg Cluster 1 vs.**  **TLE-MRIneg Cluster 2** | | **F_(1,91)_** | **t-value** | ***P*_FDR_** |
| --- | --- | --- | --- | --- |
| *Ipsilateral lateral ventriculus* | | 7.371 | -2.717 | **0.044** |
| *Ipsilateral nucleus accumbens* | | 6.234 | 2.498 | **0.044** |
| *Contralateral lateral ventriculus* | | 4.659 | -2.159 | **0.048** |
| *Contralateral nucleus accumbens* | | 6.894 | 2.628 | **0.044** |
| *Contralateral caudate* | | 4.769 | 2.185 | **0.048** |
| *Contralateral putamen* | | 5.411 | 2.327 | **0.044** |
| *Contralateral pallidum* | | 5.567 | 2.359 | **0.044** |
| *Contralateral amygdala* | |  |  |  |
|  | Lateral nucleus | 4.169 | 2.039 | 0.066 |
|  | Basal nucleus | 6.616 | 2.573 | **0.027** |
|  | AB nucleus | 6.946 | 2.634 | **0.027** |
|  | AAA | 3.746 | 1.935 | 0.075 |
|  | Central nucleus | 0.013 | 0.113 | 0.910 |
|  | Medial nucleus | 0.552 | 0.743 | 0.551 |
|  | Cortical nucleus | 8.025 | 2.834 | **0.027** |
|  | CAT | 7.226 | 2.689 | **0.027** |
|  | Paralaminar nucleus | 4.746 | 2.181 | 0.055 |
|  | BLA | 6.568 | 2.564 | **0.027** |
|  | CMA | 0.250 | 0.500 | 0.675 |
|  | Whole amygdala | 6.322 | 2.513 | **0.027** |

**Supplementary Table 9**. Subcortical volume comparisons between TLE-MRIneg Cluster 1 and Cluster 2. *Values represent MANCOVA F-statistics and t-values for subcortical comparisons between Cluster 1 and Cluster 2. Only p-values surviving false discovery rate (FDR) correction (P_FDR_ < 0.05) are reported, except for the amygdala, for which all subnuclei are shown. Significant results are highlighted in BOLD. All comparisons were adjusted for age, sex, and intracranial volume (ICV).*

| **a) TLE-MRIneg** **Cluster 1** | | | | | | | | |
| --- | --- | --- | --- | --- | --- | --- | --- | --- |
|  | **Age** | **Age of onset** | **Duration** | **Seizure frequency** | **N° ASM** | **N° past ASM** | **N° of auras** | **N° of automatisms** |
| Lateral nucleus | 0.132 | 0.152 | -0.072 | 0.027 | 0.004 | -0.248 | -0.016 | 0.167 |
| Basal nucleus | 0.160 | 0.126 | 0.019 | -0.057 | 0.027 | -0.254 | -0.148 | 0.245 |
| Paralaminar nucleus | 0.182 | 0.194 | -0.068 | -0.053 | -0.091 | -0.278 | -0.154 | 0.218 |
| BLA | 0.134 | 0.122 | -0.015 | -0.015 | 0.003 | -0.254 | -0.123 | 0.226 |
| Whole amygdala | 0.088 | 0.075 | -0.003 | 0.000 | 0.037 | -0.247 | -0.102 | 0.229 |
| **b) TLE-MRIneg Cluster 2** | | | | | | | | |
|  | **Age** | **Age of onset** | **Duration** | **Seizure frequency** | **N° ASM** | **N° past ASM** | **N° of auras** | **N° of automatisms** |
| Ipsilateral cortical thickness | -0.209 | -0.214 | 0.078 | 0.367 | 0.383 | -0.055 | -0.007 | -0.066 |
| Contralateralcortical thickess | -0.295 | -0.319 | -0.156 | **0.404*** | **0.392*** | -0.065 | 0.006 | 0.048 |

**Supplementary Table 10**. Correlations between brain measures and clinical variables within TLE-MRIneg clusters. *Values are presented as Pearson’s r. Asterisks (*) indicate correlations surviving false discovery rate (FDR) correction (P_FDR_ < 0.05). The variable “N° of auras” includes epigastric, auditory, visual, olfactory, gustatory, autonomic, psychic, and sensory-motor auras (both unilateral and bilateral), yielding a total score ranging from 0 to 9 based on the number of distinct aura types reported. Similarly, “N° of automatisms” encompasses oral, manual (unilateral and bilateral), and hyperkinetic automatisms, with a possible score ranging from 0 to 4.*

| **a) Cluster 1 drug-resistance (N=12) vs. drug-responsive** **(N=41)** | | |
| --- | --- | --- |
|  | **Stat. (F)** | **P-value** |
| Lateral nucleus | 0.508 | 0.479 |
| Basal nucleus | 0.270 | 0.606 |
| Paralaminar nucleus | 0.413 | 0.524 |
| BLA | 0.375 | 0.543 |
| Whole amygdala | 0.309 | 0.581 |
| **b) Cluster 1 with FBTCS (N=35) vs. without FBTCS (N=18)** | | |
| Lateral nucleus | 1.578 | 0.215 |
| Basal nucleus | 0.524 | 0.473 |
| Paralaminar nucleus | 1.275 | 0.264 |
| BLA | 1.202 | 0.278 |
| Whole amygdala | 1.341 | 0.253 |
| **c) Cluster 2 drug-resistance (N=12) vs. drug-responsive** **(N=31)** | | |
| Ipsilateral cortical thickness | 0.000 | 0.985 |
| Contralateral cortical thickness | 0.019 | 0.890 |
| **d) Cluster 2 with FBTCS (N=23) vs. without FBTCS (N=20)** | | |
| Ipsilateral cortical thickness | 2.776 | 0.104 |
| Contralateral cortical thickness | 1.227 | 0.275 |

**Supplementary Table 11**. Group comparisons within TLE-MRIneg clusters based on drug-resistance and FBTCS history. *Panel (a) reports ANCOVA results (F values) for ipsilateral amygdala volume comparisons between drug-resistant and drug-responsive patients within Cluster 1. Panel (b) shows the ANCOVA results for ipsilateral amygdala volume comparisons in Cluster 1 patients with and without focal to bilateral tonic-clonic seizures (FBTCS). All Cluster 1 analyses included age, sex, and intracranial volume (ICV) as covariates. Panel (c) presents ANCOVA results for bilateral mean cortical thickness comparisons between drug-resistant and drug-responsive patients within Cluster 2. Panel (d) displays the corresponding comparisons between Cluster 2 patients with and without FBTCS. Age and sex were included as covariates in all Cluster 2 analyses.*

**Supplementary Figure 8. Multivariate association between clinical features and cortical atrophy in TLE-MRIneg Cluster 2.** Partial least squares (PLS) analysis was used to examine associations between clinical features and cortical thickness in patients with MRI-negative temporal lobe epilepsy (TLE-MRIneg), Cluster 2. Latent variables (LVs) were obtained via singular value decomposition of the brain–behavior correlation matrix. Only LV-1 accounted for significantly more covariance than expected under the null model (*P*_perm_ < 0.05), explaining 35% of the total covariance (*P*_perm_ = 0.0004, top left). LV-1 loadings indicate the contribution of each clinical variable. Red bars represent bootstrap ratios; error bars indicate bootstrap-estimated standard errors (top right). Cortical regions contributing to LV-1 were identified using bootstrap ratios thresholded at ±1.96, revealing widespread cortical thinning, especially in temporo-occipital regions (bottom left). Patients exhibiting this atrophic pattern were characterized by older age, later epilepsy onset, shorter disease duration, lower seizure frequency, fewer antiseizure medications (ASMs), good treatment response, absence of family history or febrile seizures, fewer automatisms, and preserved awareness during seizures. The correlation between brain and clinical LV-1 scores was strong (r = 0.680), indicating a robust brain–behavior association (bottom center). However, the cross-validated out-of-sample correlation did not reach statistical significance (r = −0.132 vs permuted null r = −0.155, *P*_perm_ = 0.210, bottom right). FBTCS: focal to bilateral tonic-clonic seizures, SE: status epilepticus.

**Supplementary references**

1.	Bernasconi A, Cendes F, Theodore WH, et al. Recommendations for the use of structural magnetic resonance imaging in the care of patients with epilepsy: A consensus report from the International League Against Epilepsy Neuroimaging Task Force. *Epilepsia*. Published online May 28, 2019:epi.15612. doi:10.1111/epi.15612

2.	Fischl B. FreeSurfer. *NeuroImage*. 2012;62(2):774-781. doi:10.1016/j.neuroimage.2012.01.021

3.	Marcus DS, Harms MP, Snyder AZ, et al. Human Connectome Project Informatics: quality control, database services, and data visualization. *Neuroimage*. 2013;80:10.1016/j.neuroimage.2013.05.077. doi:10.1016/j.neuroimage.2013.05.077

4.	Glasser MF, Sotiropoulos SN, Wilson JA, et al. The Minimal Preprocessing Pipelines for the Human Connectome Project. *Neuroimage*. 2013;80:105-124. doi:10.1016/j.neuroimage.2013.04.127

5.	Marcus D, Harwell J, Olsen T, et al. Informatics and Data Mining Tools and Strategies for the Human Connectome Project. *Front Neuroinform*. 2011;5. doi:10.3389/fninf.2011.00004

6.	Royer J, Larivière S, Rodriguez-Cruces R, et al. Cortical microstructural gradients capture memory network reorganization in temporal lobe epilepsy. *Brain*. 2023;146(9):3923-3937. doi:10.1093/brain/awad125

7.	Iglesias JE, Augustinack JC, Nguyen K, et al. A computational atlas of the hippocampal formation using ex vivo , ultra-high resolution MRI: Application to adaptive segmentation of in vivo MRI. *NeuroImage*. 2015;115:117-137. doi:10.1016/j.neuroimage.2015.04.042

8.	Saygin ZM, Kliemann D, Iglesias JE, et al. High-resolution magnetic resonance imaging reveals nuclei of the human amygdala: manual segmentation to automatic atlas. *NeuroImage*. 2017;155:370-382. doi:10.1016/j.neuroimage.2017.04.046

9.	Iglesias JE, Insausti R, Lerma-Usabiaga G, et al. A probabilistic atlas of the human thalamic nuclei combining ex vivo MRI and histology. *arXiv:180608634 [physics, q-bio]*. Published online June 22, 2018. Accessed March 18, 2022. http://arxiv.org/abs/1806.08634

10.	Ballerini A, Biagioli N, Carbone C, et al. Late-onset temporal lobe epilepsy: insights from brain atrophy and Alzheimer’s disease biomarkers. *Brain*. 2025;148(1):185-198. doi:10.1093/brain/awae207

11.	Ballerini A, Tondelli M, Talami F, et al. Amygdala subnuclear volumes in temporal lobe epilepsy with hippocampal sclerosis and in non-lesional patients. *Brain Commun*. 2022;4(5):fcac225. doi:10.1093/braincomms/fcac225

12.	Ballerini A, Talami F, Molinari MA, et al. Exploring the relationship between amygdala subnuclei volumes and cognitive performance in left-lateralized temporal lobe epilepsy with and without hippocampal sclerosis. *Epilepsy & Behavior*. 2023;145:109342. doi:10.1016/j.yebeh.2023.109342

13.	Micalizzi E, Ballerini A, Giovannini G, et al. The role of the amygdala in ictal central apnea: insights from brain MRI morphometry. *Ann Clin Transl Neurol*. 2024;11(1):121-132. doi:10.1002/acn3.51938

14.	Price JL, Russchen FT, Amaral DG. *The Limbic Region. II: The Amygdaloid Complex.* Elsevier Sci.; 1987.

15.	Benarroch EE. The amygdala: Functional organization and involvement in neurologic disorders. *Neurology*. 2015;84(3):313-324. doi:10.1212/WNL.0000000000001171

16.	Yaniv Z, Lowekamp BC, Johnson HJ, Beare R. SimpleITK Image-Analysis Notebooks: a Collaborative Environment for Education and Reproducible Research. *J Digit Imaging*. 2018;31(3):290-303. doi:10.1007/s10278-017-0037-8

17.	Carré A, Klausner G, Edjlali M, et al. Standardization of brain MR images across machines and protocols: bridging the gap for MRI-based radiomics. *Sci Rep*. 2020;10(1):12340. doi:10.1038/s41598-020-69298-z

18.	Larivière S, Paquola C, Park B yong, et al. The ENIGMA Toolbox: multiscale neural contextualization of multisite neuroimaging datasets. *Nat Methods*. 2021;18(7):698-700. doi:10.1038/s41592-021-01186-4

19.	Glasser MF, Coalson TS, Robinson EC, et al. A multi-modal parcellation of human cerebral cortex. *Nature*. 2016;536(7615):171-178. doi:10.1038/nature18933

20.	Benjamini Y, Hochberg Y. Controlling the False Discovery Rate: A Practical and Powerful Approach to Multiple Testing. *Journal of the Royal Statistical Society: Series B (Methodological)*. 1995;57(1):289-300. doi:10.1111/j.2517-6161.1995.tb02031.x

21.	Kebets V, Holmes AJ, Orban C, et al. Somatosensory-Motor Dysconnectivity Spans Multiple Transdiagnostic Dimensions of Psychopathology. *Biol Psychiatry*. 2019;86(10):779-791. doi:10.1016/j.biopsych.2019.06.013

22.	Zöller D, Sandini C, Karahanoğlu FI, et al. Large-Scale Brain Network Dynamics Provide a Measure of Psychosis and Anxiety in 22q11.2 Deletion Syndrome. *Biol Psychiatry Cogn Neurosci Neuroimaging*. 2019;4(10):881-892. doi:10.1016/j.bpsc.2019.04.004

23.	McIntosh AR, Mišić B. Multivariate statistical analyses for neuroimaging data. *Annu Rev Psychol*. 2013;64:499-525. doi:10.1146/annurev-psych-113011-143804

24.	Krishnan A, Williams LJ, McIntosh AR, Abdi H. Partial Least Squares (PLS) methods for neuroimaging: a tutorial and review. *Neuroimage*. 2011;56(2):455-475. doi:10.1016/j.neuroimage.2010.07.034
